# Integration of Transcriptome and Exome Genotyping Identifies Significant Variants with Autism Spectrum Disorder

**DOI:** 10.3390/ph15020158

**Published:** 2022-01-27

**Authors:** Noor B. Almandil, Abdulla AlSulaiman, Sumayh A. Aldakeel, Deem N. Alkuroud, Halah Egal Aljofi, Safah Alzahrani, Aishah Al-mana, Asma A. Alfuraih, Majed Alabdali, Fahd A. Alkhamis, Sayed AbdulAzeez, J. Francis Borgio

**Affiliations:** 1 Department of Clinical Pharmacy Research, Institute for Research and Medical Consultations (IRMC), Imam Abdulrahman Bin Faisal University, Dammam 31441, Saudi Arabia; nbalmandil@iau.edu.sa; 2 Department of Neurology, College of Medicine, Imam Abdulrahman Bin Faisal University, Dammam 31441, Saudi Arabia; aalsulaiman@iau.edu.sa (A.A.); mmalabdali@iau.edu.sa (M.A.); fkhamis@iau.edu.sa (F.A.A.); 3 Department of Genetic Research, Institute for Research and Medical Consultations (IRMC), Imam Abdulrahman Bin Faisal University, Dammam 31441, Saudi Arabia; sumayh1427@gmail.com (S.A.A.); dalkroud@gmail.com (D.N.A.); asmaalfuraih95@gmail.com (A.A.A.); asayed@iau.edu.sa (S.A.); 4 Environmental Health Research Area, Institute for Research and Medical Consultations (IRMC), Imam Abdulrahman Bin Faisal University, Dammam 31441, Saudi Arabia; healjofi@iau.edu.sa; 5 Department of Mental Health, College of Medicine, Imam Abdulrahman Bin Faisal University, Dammam 31441, Saudi Arabia; sialzhrani@iau.edu.sa (S.A.); amalmana@iau.edu.sa (A.A.-m.); 6 King Fahad Hospital of the University, Imam Abdulrahman Bin Faisal University, Dammam 34212, Saudi Arabia

**Keywords:** autism spectrum disorder, neurodevelopmental disorder, SNPs, regulatory variants, gene expression, RNA sequencing

## Abstract

Autism is a complex disease with genetic predisposition factors. Real factors for treatment and early diagnosis are yet to be defined. This study integrated transcriptome and exome genotyping for identifying functional variants associated with autism spectrum disorder and their impact on gene expression to find significant variations. More than 1800 patients were screened, and 70 (47 male/23 female) with an average age of 7.56 ± 3.68 years fulfilled the DSM-5 criteria for autism. Analysis revealed 682 SNPs of 589 genes significantly (*p* < 0.001) associated with autism among the putative functional exonic variants (*n* = 243,345) studied. Olfactory receptor genes on chromosome 6 were significant after Bonferroni correction (α = 0.05/243345 = 2.05 × 10^−7^) with a high degree of linkage disequilibrium on 6p22.1 (*p* = 6.71 × 10^−9^). The differentially expressed gene analysis of autistic patients compared to controls in whole RNA sequencing identified significantly upregulated (foldchange ≥0.8 and *p*-value ≤ 0.05; *n* = 125) and downregulated (foldchange ≤−0.8 and *p*-value ≤ 0.05; *n* = 117) genes. The integration of significantly up- and downregulated genes and genes of significant SNPs identified regulatory variants (rs6657480, rs3130780, and rs1940475) associated with the up- (*ITGB3BP*) and downregulation (*DDR1* and *MMP8*) of genes in autism spectrum disorder in people of Arab ancestries. The significant variants could be a biomarker of interest for identifying early autism among Arabs and helping to characterize the genes involved in the susceptibility mechanisms for autistic subjects.

## 1. Introduction

Autism spectrum disorder (ASD) is a complex neurodevelopmental disorder that exhibits a wide range of symptoms of varying severity and has a high prevalence in the Gulf region [1]. Recent research suggests that gene variants, altered biological mechanisms, and the environment together influence the development of autism [2]. The most common symptoms of autism are impaired social skills, difficulty with communication, and repetitive movements. Autism can be detected at the age of 3 years, and perhaps as early as 18 months [3]. ASD heritability has been estimated to be 40–90%, and a number of common variants contribute to the significant variability in the condition, in addition to epigenetic, environmental, and hormonal factors [4,5,6]. Moreover, the Diagnostic and Statistical Manual of Mental Disorders—Fifth Edition includes atypical sensory processing, which has been reported in more than 70% of patients with ASD as a diagnostic criterion (DSM-5; American Psychiatric Association, 2013) [7,8,9].

Genes differentially expressed in autism are important for understanding the associated pathways [10,11,12]. This meta-analysis looked at ten Gene Expression Omnibus datasets, which included 364 cases and 248 controls. It discovered 3105 genes that were consistently expressed differently in autism (1680 downregulated genes and 1425 upregulated genes). *PNPLA2, LYPLA2, LYPLA2P1, PLA2G4D, PLA2G6, PLA2G7*, and *PLA2G5* were also discovered to be related to phospholipase A2 (PLA2). The enriched gene ontology terms for molecular functions were involved in structural constituents of ribosomes and transcription regulator activity, while the enriched gene ontology terms for biological processes were involved in translational elongation and the response to cytokine stimuli, according to the study. The ribosome route was the most important pathway in our KEGG study. The meta-analysis discovered genes that were consistently differentially expressed in autism, as well as biological pathways linked to gene expression alterations [10]. Topological analysis of the autistic brain identified proteomic hub gene signatures: *CDK2*, *BAG3*, *MYC*, *TP53*, *HDAC1*, *CDKN1A*, *EZH2*, *GABARAPL1*, *TRAF1*, and *VIM* [11]. The study also revealed the transcriptional regulating factors, such as *YY1*, *FOXL1*, *USF2*, *FOXC1*, *GATA2, NFIC*, *E2F1*, *NFKB1*, *TFAP2A*, and *HINFP* [11].

Despite rapid developments in genetic techniques, for diagnostic purposes, common variants of neuropsychiatric disorders are still lacking. An exome microarray provides a potential diagnostic method for many complex genetic diseases such as ASD. Unlike other genetic techniques, the exome microarray is effective for profiling variants in associated genes with >5% allele frequency [13]. In addition, it provides exceptional coverage of the whole exonic region delivered from over 12,000 individual exome sequences [14]. The present study conducted whole RNA sequencing and exome microarray analysis to identify the most prominent variants associated with autism in patients in Saudi Arabia.

## 2. Results

**Figure 1:**
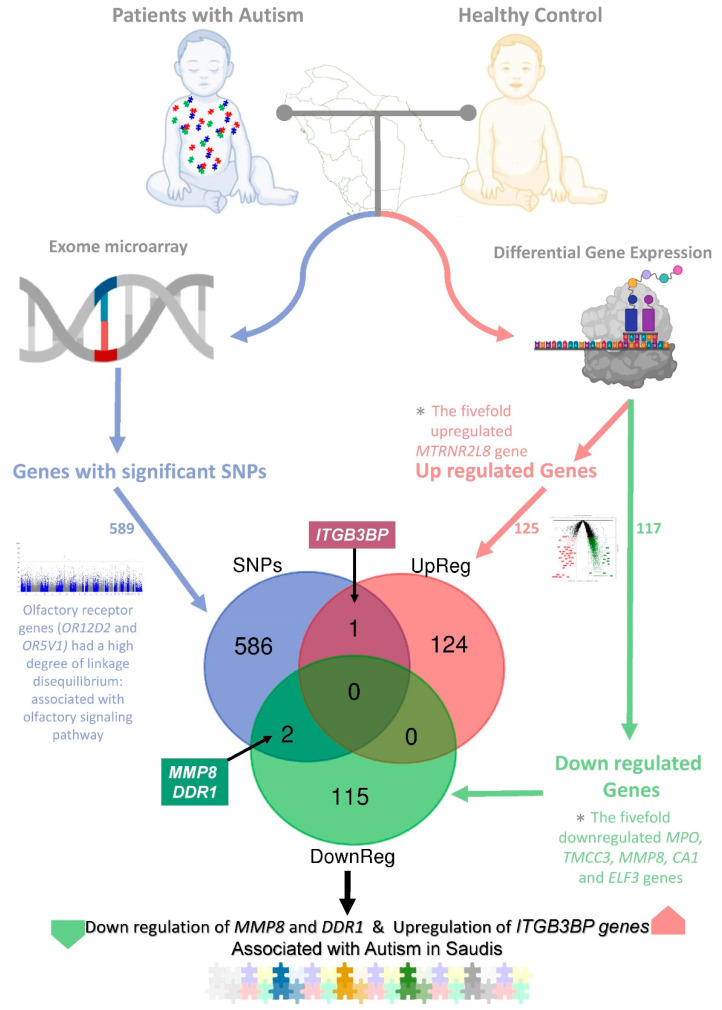
Integration of whole RNA sequencing-based differentially expressed gene analysis (upregulated: foldchange ≥0.8 and *p*-value ≤ 0.05, *n* = 125; downregulated: foldchange ≤−0.8 and *p*-value ≤ 0.05, *n* = 117) of patients with autism in relation to the healthy controls with genes of significant genotypes. Venn diagram analysis of the curated data identifies three (rs6657480, rs3130780, and rs1940475) regulatory variants in the up- (*ITGB3BP*) and downregulated (*DDR1* and *MMP8*) genes in autistic patients. * List of the fivefold downregulated and upregulated genes can be considered for the early identification of autism patients among Saudis.

The study revealed the significant regulatory variants associated with the development of autism in people with Arab ancestries using multiomic analysis (Figure 1).

**Figure 2:**
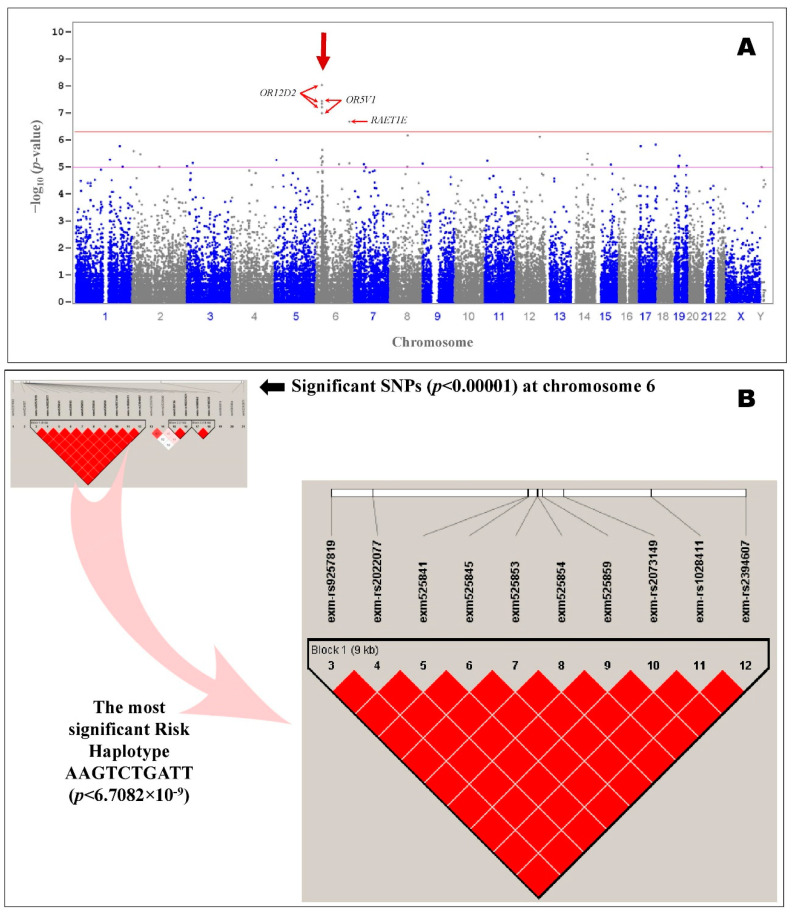
(**A**): Manhattan plot of putative functional exonic variants *n* = 243,345 in the molecular genetics of autism from the association study. The log10 (*p*) values from the association are plotted according to variant position on each chromosome. Positions of candidate genes of functional exonic variants at olfactory receptor genes on chromosome 6p22.1 for autism are indicated by red arrows. The horizontal red line indicates the preset threshold of *p*= 4.00 × 10^−7^. The horizontal pink line indicates the suggestive threshold of *p* = 1.00 × 10^−5^. (**B**): Genetic association of the most significant variants at the *OR12D2* and *OR5V1* genes in the chromosome 6p22.1 region and linkage disequilibrium results in subjects of Arab ancestry with autism. Block 1 indicates the most significant risk haplotype, AAGTCTGATT (*p* = 6.7082 × 10^−9^), associated with the Arab-ancestry autism subjects.

A total of 13 SNPs were significantly (*p* < 2.05 × 10^−7^) associated with ASD even after Bonferroni or false discovery rate corrections (corrected α = 0.05/243,345 = 2.05 × 10^−7^) among the 243,345 SNPs screened, and they obeyed the HWE. Table 1 summarizes the most significant SNPs that were associated with autism in patients of Arab ancestry. Common variants in olfactory receptor family 12 subfamily D member 2 (*OR12D2*) and olfactory receptor family 5 subfamily V member 1 (*OR5V1*) showed the strongest signal in Arab patients with autism. The top 10 most significantly (*p* < 2.05 × 10^−7^) associated SNPs were in the region of the olfactory receptor genes *OR12D2* and *OR5V1* on chromosome 6p22.1 (Figure 2). Additional information about the significant SNPs (*p* < 0.0001) in autism patients is provided in Table S1. The results suggest that a single gene, *OR12D2* (including up and downstream; NC_000006.11:g.29360183–g.29369519), has 10 common SNPs with large effects on risk (rs9257819, rs2022077, rs9257834, rs4987411, rs2073154, rs2073153, rs2073151, rs2073149, rs1028411, and rs2394607), including five SNPs in the coding region (rs9257834, rs4987411, rs2073154, rs2073153, and rs2073151).

All the association tests were screened using the frequency of minor alleles in controls, the *p*-value of the HWE, and the type I error rate to identify the strongest genetic findings, which were input for linkage disequilibrium in HapMap SNPs of selected regions on chromosome 6 (Figure 2). The variants in the region of the olfactory receptor genes *OR12D2* and *OR5V1* had a high degree of linkage disequilibrium (*p* = 6.71 × 10^−9^) (Figure 2). The haplotype associated with the most significant risk in patients of Arab ancestry with autism, AAGTCTGATT (*p* = 6.7082 × 10^−9^), consisted of the top 10 SNPs (rs9257819A, rs2022077A, rs9257834G, rs4987411T, rs2073154C, rs2073153T, rs2073151G, rs2073149A, rs1028411T, and rs2394607T) (Table 2). The protective haplotype most significantly associated with the control subjects of Arab ancestry, CTTCGGATGC (*p* = 2.43 × 10^−8^), also consisted of the top 10 SNPs (rs9257819C, rs2022077T, rs9257834T, rs4987411C, rs2073154G, rs2073153G, rs2073151A, rs2073149T, rs1028411G, and rs2394607C) with the alleles that were not associated with autism (Table 2).

### 2.1. RNA Sequencing and Differentially Expressed Genes

**Figure 3:**
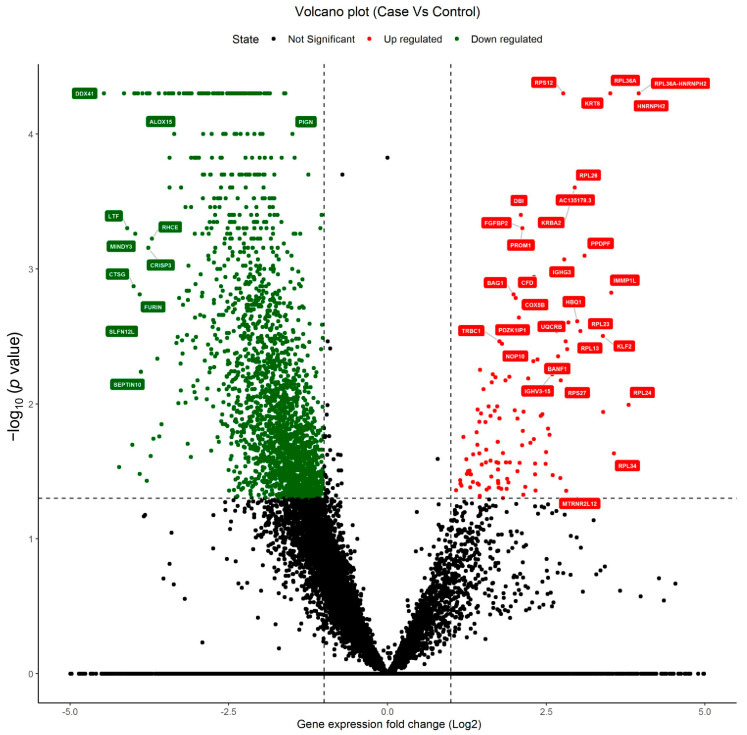
Volcano plots of the significantly upregulated genes (red colored) and downregulated genes (green colored) between autism and controls. Upregulated: foldchange ≥ 0.8 and *p*-value ≤ 0.05; downregulated: foldchange ≤ −0.8 and *p*-value ≤ 0.05.

Blood samples were collected in the RNA protectant vacutainer with an RNA stabilizer to obtain more accurate results. Total RNA was isolated for the transcriptome profiling study using paired-end sequencing. Raw reads were checked for a quality Phred score ≥ 30. The qualifying reads were processed for mapping on the reference genome and expression profile preparation. The raw RNA sequencing data reads were trimmed for the adaptors and mapped by using the tophat RNA Sequence mapping tool with the reference RNA sequence. All RNA sequence data were subjected to complete differential expression analysis between the autistic and the control subjects. The differential expression between the autistic and control subjects was calculated, revealing upregulated (foldchange ≥ 0.8 and *p*-value ≤ 0.05) and downregulated (foldchange ≤ −0.8 and *p*-value ≤ 0.05) genes. Autistic patients and healthy controls were considered two distinct groups for differential expression calculation using Cuffdiff [15]. Genes were filtered for differential expression through all significant DEGs based on a *p*-value ≤ 0.05. For upregulated genes, we used a cutoff *p*-value ≤ 0.05 and foldchange ≥ 0.8, and for downregulated genes, we used a cutoff *p*-value ≤ 0.05 and foldchange ≤ −0.8. All the expressed genes from the autistic and control samples with a quality Phred score ≥ 30 were subjected to differential expressed gene analysis, and the results are presented in Figure 3 as volcano plots. The significantly upregulated and downregulated genes were considered with foldchange ≥ 0.8 and *p*-value ≤ 0.05 and foldchange ≤−0.8 and *p*-value ≤ 0.05, respectively.

A list of the top 20 upregulated genes with foldchange ≥ 1.98, a *p*-value ≤ 0.0025 and a *q*-value ≤ 0.05 is presented in Table 3. A list of the top 20 downregulated genes with foldchange ≤ −3.5, a *p*-value ≤ 0.00005, and a *q*-value ≤ 0.0044 is presented in Table 4. The results indicate that the fivefold downregulated genes (foldchange ≤ −5, *p*-value ≤ 0.00005 and *q*-value ≤ 0.0044) such as *MPO*, *TMCC3*, *MMP8*, *CA1*, and *ELF3* can be notable downregulation biomarkers. Similarly, a fivefold upregulated gene, *MTRNR2L8* (foldchange ≥ 1.98, *p*-value ≤ 0.00005 and *q*-value ≤ 0.004), is a considerable upregulation biomarker. The fivefold downregulated *MPO*, *TMCC3*, *MMP8*, *CA1*, and *ELF3* genes and the fivefold upregulated *MTRNR2L8* gene can be considered for the early identification of autistic patients among Saudis.

### 2.2. Pathway Enrichment Analysis

**Figure 4:**
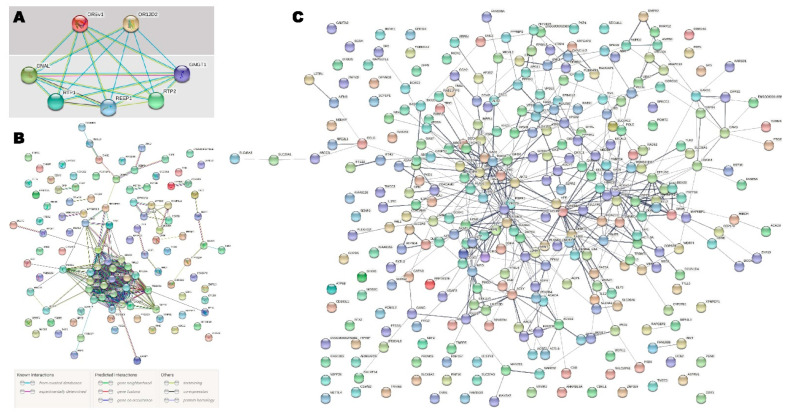
(**A**): Protein–protein interaction based on the predictive model and predicted functional partners of the OR12D2 and OR5V1 proteins using the STRING database, version 10. (**B**): Significantly upregulated genes and their interaction. Number of edges: 77 and the PPI enrichment *p*-value <1.0×10^−16^. (**C**): Significantly downregulated genes (*p*-value <0.001) and their interaction. Number of edges: 420 and the PPI enrichment *p*-value = 2.05×10^−06^.

All the significantly upregulated and downregulated genes were searched for during the GO and pathway enrichment analysis. Significantly upregulated genes and their interactions (Figure 4) revealed highly significant protein–protein interactions based on the predictive model enrichment (*p*-value: <1.0 × 10^−16^). Downregulated genes (*p*-value < 0.001) and their interactions (Figure 4) demonstrated significant PPI enrichment with a *p*-value = 2.05 × 10^−6^. Pathway enrichment by the significantly upregulated and downregulated genes is presented in Table 5. Ribosome, oxidative phosphorylation, protein export, Parkinson’s disease, and Alzheimer’s disease-associated pathways were significantly enriched by the upregulated genes (Table 5). The impact on the genetic information processing and translation was significantly enriched by the 17 upregulated genes through the ribosome pathway. The HIF-1 signaling pathway, pyruvate metabolism, propanoate metabolism, metabolic pathways, the rap1 signaling pathway, the AMPK signaling pathway, the glucagon signaling pathway, proteoglycans in cancer, renal cell carcinoma, fatty acid biosynthesis, the insulin signaling pathway, and EGFR tyrosine kinase inhibitor resistance were significantly enriched by the downregulated genes (Table 5).

### 2.3. Integration of Transcriptome and Exome Genotyping

Genotyping and exome array analysis among the 243,345 putative functional exonic variants revealed 589 genes significantly (*p* < 0.001) associated with autism, with 682 significant variants. Furthermore, the differentially expressed gene analysis based on transcriptome sequencing for the autistic patients compared to controls identified significantly upregulated (foldchange ≥0.8 and *p*-value ≤ 0.05; *n* = 125) and downregulated (foldchange ≤−0.8 and *p*-value ≤ 0.05; *n* = 117) genes. The integration of significantly up- and downregulated genes and genes of significant SNPs using Venn diagram analysis identified three regulatory variants (rs6657480, rs3130780, and rs1940475) associated with upregulated (rs6657480, *p* = 0.000762, (NC_000001.10: g.63999868T > C) nearest upstream to the gene *ITGB3BP*) and downregulated (rs3130780, *p* = 3.12 × 10^−5^ (NC_000006.11: g.30874308T > G) nearest upstream gene, *DDR1;* and rs1940475, *p* = 0.0001807 (NC_000011.9:g.102593248T > C; NM_002424.3:c.259A > G; NP_002415.1:p.Lys87Glu, *MMP8*)) genes in autism spectrum disorder in patients of Arab ancestries (Figure 1).

## 3. Discussion

This study performed a comprehensive investigation of the genetic architecture of ASD using an exome microarray analysis and RNA sequencing. We identified 155 significantly (*p* < 0.0001) associated SNPs, and the most strongly (*p* < 2.05 × 10^−7^) associated 13 SNPs were investigated further. None of the 13 SNPs had previously been reported in Saudis with autism [16]. The *OR12D2* olfactory receptor gene, which codes for the G-protein coupled receptor (GPCR), carries the signal to sensory neurons to trigger smell and has 10 common variants and large effects on risk, was associated with autism in Arab-ancestry subjects and showed a high degree of linkage disequilibrium (*p* = 6.71 × 10^−9^) on the significant risk haplotype AAGTCTGATT. The *OR12D2* gene (gene expression for *OR12D2:* ENSG00000168787.4) was overexpressed in the brain (hypothalamus ×13.9, caudate ×10.7, hippocampus ×8.2, nucleus accumbens ×5.0 and cervix-endocervix ×4.6) [17,18].

Some studies have proposed unusual sensory processing, particularly olfactory processing, in a number of neurodevelopmental disorders, including autism [19,20]. Berko et al. [21] showed that the *OR2L13* G-protein, joined by the olfactory receptor, which is involved in resetting the neuronal response to smells, is particularly prevalent in autism in terms of DNA methylation and expression, suggesting a possible rationale for olfactory dysfunction in ASD. The olfactory receptor genes *OR12D2* and *OR5V1*, which are involved in the olfactory signaling pathway, G alpha (s) signaling events, GPCR downstream signaling, and GPCR signaling and signal transduction together regulate the overall olfactory growth, behavior, and impairment associated with autism (Figure 4) [22]. A functional analysis of the genes is presented in Table S2. The significant genes in this study included integrin subunit alpha 11 (*ITGA11*), which is associated with attention deficit hyperactivity disorder and attention deficit disorder with hyperactivity; MHC class I polypeptide-related sequence A (*MICA*), associated with symptoms of inflammatory bowel disease, chronic ulcerative colitis, and Crohn’s disease; bromodomain-containing 2 (BRD2), which has been linked to Alzheimer’s disease and myoclonic epilepsy; cell division cycle 14C pseudogene (*CDC14C*), which is associated with Parkinson’s disease and sleep; methylenetetrahydrofolate dehydrogenase (NADP+ dependent) 1-like (*MTHFD1L*), which is associated with stroke and Alzheimer’s disease; myosin IXB (*MYO9B*), associated with symptoms of celiac disease, inflammatory bowel disease, chronic ulcerative colitis, multiple sclerosis, and schizophrenia; and ring finger protein 144A (*RNF144A*), which has been associated with depressive disorder, schizophrenia, and stroke (Table S2) [23,24,25,26,27,28,29,30,31,32,33,34,35]. For example, SNP rs2727943 is significantly correlated with ASD and has been reported in bipolar cases as an intergenic variant that occurs between the *BIG-2* and *CNTN6* genes, both of which produce a neuronal membrane protein that forms the axonal connections in the developing nervous system [4].

Unlike previous results, our study identified a single gene, *OR12D2* (including up and downstream; NC_000006.11:g.29360183-g.29369519), with 10 common SNPs and large effects on risk in patients with autism. Most previous results have suggested that no single gene has such large effects on risk (*p* = 6.71 × 10^−9^) [4]. A comprehensive review on the genetics of ASD described the role of various genes and their clinical relevance to autism. Here, we describe an additional gene, *OR12D2*, that should be considered for molecular pathway studies in the future [4]. The enrichment analysis of the genes/nearest upstream/nearest downstream genes of the highly associated SNPs using Enrichr in GWAS Catalogue 2019 revealed significant diseases/drug terms associated with ASD or schizophrenia (*p* = 0.001107; adjusted *p* = 0.0321) on eight genes, namely *SFTA2*, *BRD2*, *HCP5*, *OR5V1*, *HLA-DMA*, *ZKSCAN8*, *OR12D2*, and *MICA* [36]. Four genes (*TMEM185B*, *MTHFD1L*, *QPCT*, and *H2AFV*, *p* = 0.0098) were downregulated relative to their median expression in the ventral juxtacommissural pretectal nucleus among the genes/nearest upstream/nearest downstream genes of highly associated SNPs using Enrichr [36]. Children with systemic lupus erythematosus (SLE) have a high risk of developing autism. In the present study, seven genes (*BRD2*, *OR12D2*, *CR2*, *OR5V1*, *SFTA2*, *HCP5*, and *HLA-DMA*) were highly significant for SLE (*p* = 0.000029; Bonferroni = 0.0041) using DAVID [37,38].

The risk haplotype of the *OR12D2* gene in autism subjects and the overexpression of *OR12D2* in the brain, dysfunction in neurodegenerative processes, olfactory impairment, and the overuse of aroma compounds or essential oils, flowers, aromatic wood, herbs, frankincense, and perfumes among the Arab population provide clues that olfactory sensory deficits and overexpression of *OR12D2* should be studied in detail among the Arab population to reveal the underlying etiology of autism [18,19,20,21,39,40].

Even though the olfactory receptor genes on chromosome 6 are significant after a Bonferroni correction (α = 0.05/243345 = 2.05 × 10^−7^) with high linkage disequilibrium (*p* = 6.71 × 10^−9^ on 6p22.1), their significance on the transcriptome sequencing was lacking. Furthermore, the most significantly upregulated gene, *RPS12*, and the most significant downregulated gene, *MPO*, were observed with no significant variants in the exome array. This suggests that whole-genome sequencing on these subjects may reveal the significant genes with novel variants which are not covered in the exome array. Differentially expressed genes in autism are essential for the complete understanding of the associated functional and autism development pathways [10,11,12]. Earlier studies have revealed many significantly differentially expressed genes from various populations [10,11,12]. However, there were no studies on the differentially expressed genes in autism in Saudi Arabians. Hence, this study can be considered as a first of its kind study on autistic patients from Saudi Arabia. Additionally, the integrated analysis of exome array and transcriptome sequencing is an initial study on the Arab ancestries with autism. The upregulated integrin subunit Beta 3 binding protein (*ITGB3BP*) gene with rs6657480 (*p* = 0.000762) in the present study is in line with the previous studies on the genes predicted to affect risk for ASD [41]. The upregulated *ITGB3BP* gene in the present study is targeted as an autism-risk gene in human fetal brains [42], and also differential expression was observed in a mouse model [43]. Downregulation of *ITGB3BP* was observed in GABAergic neural development [44]. SNPs associated with schizophrenia risk were identified in the gene *ITGB3BP* [45].

SNP rs3130780 was observed in this study, which is located close to the downregulated discoidin domain receptor 1 (*DDR1*) gene. *DDR1* was reported earlier as a susceptibility gene for schizophrenia [46,47] and self-consciousness [48]. *DDR1*-mediated signaling systems were observed in *Drosophila* in the mushroom body encoding learning and memory [49,50]. *DDR1* plays an important role in myelination and neuropsychiatric diseases [51]. *DDR1* expression increases in parallel with neural myelination throughout mouse brain development, according to previous research [52]. The exonic variation of rs1940475 (NP_002415.1:p.Lys87Glu) in the *MMP8* gene was found to be a significant coding variant in the genotype association study, and it impacts the expression level of the *MMP8* gene. Pathway analysis and genome-wide association in major depressive disorder revealed a significant association with the *MMP8* gene [53]. rs1940475 in the *MMP8* gene was reported for its association with osteoarthritis [54], femoral head osteonecrosis [55], and bladder cancer in non-smokers [56]. On the other hand, a recent meta-analysis of the K87E (rs1940475) variant showed that this variant is not significantly associated with cancer susceptibility [57]. Overall, the literature on the *ITGB3BP*, *DDR1*, and *MMP8* genes indicates that the observation regarding the integration of transcriptome and exome genotyping in autism is a notable addition for the understanding of the genetics of autism. Defects in the SH3 and multiple ankyrin repeat domains 3 (Shank3) protein cause numerous synaptopathies, including autism [58,59,60]; upregulation of the *RPL36A* was reported in the striatal synaptosome of Shank3-overexpressing transgenic mice [58]. This is in line with the present observation on the upregulation of *RPL36A* in autistic patients and indicates that the upregulation of RPL36A can influence the development of autism through the dysfunction of SHANK3-associated synaptopathies [58,61]. The small number of subjects enrolled in this study was a notable limitation. However, the autistic patients were selected from 1800 patients visiting the neurology clinic. Currently, research guiding medication choice and dosing in patients with ASD is limited. Autism symptoms are usually managed with a variety of medications such as psychotropic (antipsychotics and antidepressants) drugs to manage behaviors such as mood symptoms, resulting in polypharmacy- and drug-related adverse effects. Knowledge of the clinical and genetic predictors of drug response variability and the outcomes of personalized medicine can advance the clinical care of young patients with ASD. Pharmacogenomics is a method that can be used for medication choice that affects a person’s genetic information and clinical presentation to make informed choices, but no studies have specifically examined the outcomes of PGX for patients with ASD. We believe that we can use the result of our study to help in the diagnosis and treatment of patients with autism. Further studies are needed to provide further information on the genetic risk in relation to treatment effectiveness. Analysis of reactive oxygen species (ROS) generation and stress enzymes in the cases and controls may strengthen the observed findings.

## 4. Materials and Methods

### 4.1. Sample Collection

This study was conducted in accordance with the Declaration of Helsinki and was approved by the Institutional Review Board (IRB) of Imam Abdulrahman Bin Faisal University. Cheek swab samples were collected for DNA and blood samples for RNA from children and adolescents up to 18 years of age from the Eastern Province of Saudi Arabia upon receiving signed informed consent from a parent and/or legal guardian for participation in the study. A total of 202 subjects of Arab ancestry (132 controls and 70 cases) provided cheek swab samples for genomic DNA extraction for exome genotyping (Table 6). A total of 1800 patients were evaluated in a pediatric neurology clinic and screened for autism from January 2018 to December 2018. Of these, 70 patients (47 males and 23 females) with an average age of 7.56 ± 3.68 years fulfilled the DSM-5 criteria for autism [9]. The clinical evaluation of the patients revealed an overall positive family history of 8.57% (*n* = 6). Patients with comorbidities were excluded from this study. Healthy subjects without familial autism were selected as controls.

### 4.2. DNA Extraction and Exome Array

The Gentra Puregene Buccal Cell Kit (Qiagen, Hilden, Germany) was used to extract DNA from the buccal cell samples. The Human Exome Bead Chip Kit v1.0 and v1.1 Illumina (San Diego, CA, USA), which consists of 243,345 putative functional exonic markers, was utilized with Illumina iScan for the microarray. All DNA samples were processed according to the manufacturer’s protocol, and iScan control software (Illumina) was used to acquire data. DNA extraction and the microarray genotyping and analysis were performed at the genetics research laboratory of the Institute for Research and Medical Consultations, Imam Abdulrahman Bin Faisal University, Dammam, Saudi Arabia. Genotyping was carried out in this lab from 2016 to 2018 using the same chip and procedures.

### 4.3. Genotyping and Functional Analysis

GenomeStudio 2.0 Data Analysis software (Illumina) was used for the initial quality check of the call rate. A total of 21 controls and 2 patients with autism were excluded from the analysis due to a call rate <0.975%, and the results were thus re-clustered. The Hardy–Weinberg equilibrium (HWE) was tested separately among the case and control groups with a 1 degree of freedom (df) genotypic chi-square test. Differences in clinical characteristics between cases and controls were calculated by the two-sample *t*-test or the χ^2^ test, as appropriate, using IBM SPSS Statistics version 23 software (IBM Co., Armonk, NY, USA). Kaviar [62] and SNP-Nexus [63] were used to confirm variants reported at a base pair position on the respective chromosome as per Genome Reference Consortium Human Build 37 (GRCh37.p13.). Case–control association analyses, odds ratios, and 95% confidence intervals were calculated to evaluate the effects of different alleles and haplotypes using Haploview version 4.2 [64] and gPLINK version 2.050 [65]. Bonferroni corrections or false discovery rate corrections were applied to correct the *p*-values of the 243,345 single nucleotide polymorphisms (SNPs) (corrected α = 0.05/243,345 = 2.05 × 10^−7^) to control inflation of the type I error rate. A *p*-value < 0.05 was considered significant. The highly significant (*p* < 1 × 10^−5^) genes were annotated for functional implications using DAVID 6.7 [37], STRING 10.5 [66], SNPnexus [63], Expression Atlas [17], Reactome [67], FunRich [68], Toppgene [69], and Enrichr [36].

### 4.4. Whole RNA Sequencing and Differentially Expressed Genes

Total ribonucleic acid (RNA) was extracted from blood (30 controls and 30 patients with autism) using the RNeasy plus micro kit (Qiagen, Hilden, Germany) following the manufacturer’s guidelines. Total RNA was quantitated using the Qubit RNA HS assay kit (Life Technologies, Eugene, OR, USA), and the quality was assessed by an Agilent 2100 (Agilent, Santa Clara, CA, USA). High-quality RNA was used to construct the cDNA library. Twenty milligrams of total RNA from each sample was treated separately with oligo (dT) for mRNA enrichment. N6 random primers were used for reverse-transcribing the target RNA fragments into double-strand cDNA. End repair was performed on double-strand cDNA fragments, and adaptors were ligated to the double-strand cDNA. All the ligated double-strand cDNA were PCR-amplified, denatured by heat into single-strand DNA and quantified, and subjected to DNA nanoball synthesis and sequencing on the DNBseq platform. Transcriptome assembly was carried out after subjecting the raw reads to quality control. Reads of adaptors and unknown bases, as well as low-quality reads, were discarded. The raw RNA sequencing data reads with high quality were mapped using the tophat RNA Sequence mapping tool. The aligned sequence data were analyzed for the differential expression between the autistic and the control subjects by considering the foldchange ≥ 0.8 and *p*-value ≤ 0.05 for upregulated genes and the foldchange ≤ −0.8 and *p*-value ≤ 0.05 for downregulated genes. All the expressed genes from the autistic and control samples with a quality Phred score ≥ 30 were only subjected to the differential expressed gene analysis. The highly significantly up- and downregulated genes were annotated for pathway enrichment analysis using DAVID 6.7 [37], STRING 10.5 [66], Expression Atlas [17], Reactome [67], FunRich [68], Toppgene [69], and Enrichr [36].

## 5. Conclusions

The Saudi autistic patients are significantly different according to the gene expression profile. The fivefold downregulated *MPO*, *TMCC3*, *MMP8*, *CA1*, and *ELF3* genes and the fivefold upregulated *MTRNR2L8* gene can be considered for the early identification of autism patients among Saudis. This study identified the *OR12D2* gene, including the 5’ and 3’ regions associated with 10 ASD SNPs on chromosome 6p22.1. The SNPs detected in patients with autism in the current study are variants in genes that either control fundamental cell survival processes or direct neurological functions that might contribute to the severity of ASD. Screening the molecular defects that are prevalent in Saudi autistic cases is a very important step to better understand the possible genes that are involved in the development of ASD. Regulatory variations (rs6657480, rs3130780, and rs1940475) identified the up- (*ITGB3BP*) and downregulation (*DDR1* and *MMP8*) of genes in autism spectrum disorder in patients of Arab ancestry discovered through the integration of up- and downregulated genes and genes of significant genotypes. Further studies are needed to investigate the pathogenic pathways of the reported SNPs and genes to develop potential therapeutic targets in the future. The presence of a panel of the most prevalent variants in ASD cases will aid in enhancing our knowledge and understanding of the complexity behind the variability in ASD.

## Figures and Tables

**Table 1:** List of the most significant SNPs associated with autistic patients.

S.No	SNP ID	CHR	BP	MA	MAF	CHISQ	P	OR (L95–U95)	Gene	AA	Case, Control Frequencies	HWpval
1	rs2073149	6	29365423	A	0.5795	33.14	8.57 × 10^−9^	0.2367 (0.143–0.3918)	*OR12D2*	A	0.754, 0.424	0.0563
2	rs2073153	6	29364835	G	0.5465	30.5	3.34 × 10^−8^	0.2489 (0.15–0.4131)	*OR12D2*	T	0.769, 0.456	0.0388
3	rs2073151	6	29364951	A	0.5398	30.1	4.10 × 10^−8^	0.2498 (0.1501–0.4155)	*OR12D2*	G	0.773, 0.457	0.0504
4	rs2394607	6	29369519	C	0.5682	30.07	4.18 × 10^−8^	0.2586 (0.1576–0.4241)	*OR5V1*	T	0.746, 0.438	0.1677
5	rs9257819	6	29360183	C	0.5398	29.55	5.46 × 10^−8^	0.2558 (0.1545–0.4235)	*OR5V1*	A	0.769, 0.462	0.0394
6	rs9257834	6	29364615	T	0.5398	29.55	5.46 × 10^−8^	0.2558 (0.1545–0.4235)	*OR12D2*	G	0.769, 0.462	0.0301
7	rs4987411	6	29364643	C	0.5398	29.55	5.46 × 10^−8^	0.2558 (0.1545–0.4235)	*OR12D2*	T	0.769, 0.462	0.0301
8	rs2073154	6	29364815	G	0.5398	29.55	5.46 × 10^−8^	0.2558 (0.1545–0.4235)	*OR12D2*	C	0.769, 0.462	0.0394
9	rs1028411	6	29367399	C	0.5398	29.55	5.46 × 10^−8^	0.2558 (0.1545–0.4235)	*OR5V1*	T	0.769, 0.462	0.0394
10	rs2022077	6	29361124	A	0.5398	28.55	9.14 × 10^−8^	0.261 (0.1575–0.4325)	*OR5V1*	A	0.766, 0.466	0.0475
11	rs9383583	6	150212003	T	0.2898	27.09	1.94 × 10^−7^	0.1395 (0.06093–0.3193)	*RAET1E*	C	0.946, 0.719	0.2627
12	rs28703878	8	79417222	G	0.233	24.82	6.30 × 10^−7^	3.396 (2.08–5.544)	*LOC105375911*	G	0.508, 0.255	0.1448
13	rs7963027	12	108894909	C	0.6118	24.58	7.15 × 10^−7^	0.3029 (0.1874–0.4894)		T	0.677, 0.436	0.9318
14	rs571264	17	74878259	A	0.3693	23.29	1.39 × 10^−6^	0.2397 (0.1307–0.4394)	*MGAT5B*	G	0.877, 0.667	0.0024
15	rs62637606	17	8172506	G	0.0511	23.09	1.55 × 10^−6^	5.81 (2.656–12.71)	*PFAS*	G	0.238, 0.062	1
16	rs2523590	6	31327064	G	0.4059	22.43	2.18 × 10^−6^	0.2661 (0.1511–0.4689)		T	0.846, 0.619	0.037
17	rs6741819	2	7147973	T	0.4602	22.27	2.37 × 10^−6^	0.2932 (0.174–0.4942)	*RNF144A*	C	0.800, 0.576	0.7589
18	rs888096	2	37603801	G	0.3466	21.74	3.12 × 10^−6^	3.016 (1.885–4.828)	*LOC107985868*	G	0.615, 0.371	1
19	rs2253705	6	30900094	A	0.125	21.38	3.76 × 10^−6^	3.706 (2.086–6.583)	*SFTA2*	T	0.346, 0.129	1
20	rs6911487	6	23774487	A	0.358	21.1	4.37 × 10^−6^	2.965 (1.853–4.743)		A	0.623, 0.386	1
21	rs4902780	14	70591661	C	0.5795	20.96	4.69 × 10^−6^	0.3342 (0.2077–0.5379)	*SLC8A3*	T	0.685, 0.414	0.0077
22	rs267733	1	150958836	G	0.0804	20.9	4.84 × 10^−6^	4.377 (2.245–8.535)	*ANXA9*	G	0.277, 0.120	0.8843
23	rs7729273	5	7228047	T	0.0454	20.8	5.10 × 10^−6^	5.765 (2.53–13.13)		T	0.215, 0.057	1
24	rs2132517	11	10791983	A	0.25	20.71	5.33 × 10^−6^	0.1707 (0.07411–0.3933)	*CTR9*	G	0.946, 0.776	0.4454
25	rs580962	6	32925692	G	0.5852	20.61	5.63 × 10^−6^	0.3383 (0.2105–0.5436)	*HLA-DMA*	T	0.677, 0.457	1
26	rs516535	6	32942302	C	0.5852	20.61	5.63 × 10^−6^	0.3383 (0.2105–0.5436)	*BRD2*	A	0.677, 0.462	1
27	rs9266825	6	31382882	A	0.4659	20.38	6.35 × 10^−6^	0.3147 (0.1885–0.5253)	*MICA*	C	0.785, 0.571	0.0328
28	rs10223421	6	31390055	T	0.4659	20.38	6.35 × 10^−6^	0.3147 (0.1885–0.5253)	*HCP5*	G	0.785, 0.571	0.0328
29	rs9854207	3	27614316	C	0.25	20.38	6.36 × 10^−6^	3 (1.848–4.869)		C	0.500, 0.262	0.09
30	rs505358	6	151327848	A	0.5909	20.27	6.72 × 10^−6^	0.3422 (0.2132–0.5492)	*MTHFD1L*	G	0.669, 0.452	0.0046
31	rs10970979	9	334337	G	0.2045	20.23	6.86 × 10^−6^	3.122 (1.883–5.176)	*DOCK8*	G	0.445, 0.214	0.3033
32	rs1802437	7	44874113	T	0.5698	20.12	7.29 × 10^−6^	0.3336 (0.2051–0.5428)	*H2AFV*	C	0.694, 0.436	0.6251
33	rs941920	14	91739081	A	0.233	20.07	7.47 × 10^−6^	0.1593 (0.06538–0.3883)	*CCDC88C*	G	0.954, 0.793	0.2034
34	rs7168069	15	68624396	A	0.233	20.07	7.47 × 10^−6^	0.1593 (0.06538–0.3883)	*ITGA11*	C	0.954, 0.795	0.4658
35	rs62620225	6	28117331	T	0.1591	19.98	7.84 × 10^−6^	0.04097 (0.005498–0.3054)	*ZKSCAN8*	C	0.992, 0.833	0.0025
36	rs1545620	19	17303774	T	0.358	19.91	8.14 × 10^−6^	2.87 (1.796–4.586)	*MYO9B*	T	0.615, 0.362	0.0766
37	rs7507442	19	53278953	A	0.6193	19.88	8.24 × 10^−6^	0.3481 (0.2176–0.5567)	*ZNF600*	G	0.638, 0.429	0.083
38	rs2727943	3	1897973	T	0.2184	19.84	8.44 × 10^−6^	0.1432 (0.05462–0.3752)		C	0.962, 0.782	0.4488
39	rs1507765	1	207535246	A	0.5795	19.74	8.87 × 10^−6^	0.3463 (0.2155–0.5562)	*CD55*	C	0.677, 0.462	0.2045
40	rs906998	8	78530715	T	0.5795	19.74	8.87 × 10^−6^	0.3463 (0.2155–0.5562)		C	0.677, 0.443	0.4206
41	rs6542573	2	120986872	T	0.3864	19.73	8.91 × 10^−6^	0.2888 (0.1641–0.508)		C	0.846, 0.629	0.0841
42	rs2633350	19	16808183	C	0.4148	19.65	9.32 × 10^−6^	0.3033 (0.1765–0.521)		T	0.823, 0.614	0.9978
43	rs1012036	7	52472450	T	0.3663	19.58	9.66 × 10^−6^	0.2781 (0.1547–0.4998)	*CDC14C*	C	0.862, 0.660	0.0124

CHR: chromosome; SNP ID: single nucleotide polymorphism ID; BP: base pair position at the respective chromosome as per GRCh37.p13; MA: minor allele name; MAF: frequency of minor allele in controls; CHISQ: basic allelic test chi-square; P: *p*-value; OR: odds ratio; SE: standard error; L95: lower bound of 95% confidence interval for odds ratio; U95: upper bound of 95% confidence interval for odds ratio. AA: associated allele; HWpval: *p*-value of Hardy–Weinberg equilibrium.

**Table 2:** Significant haplotypes of SNPs on chromosome 6 in autistic patients.

Block	Haplotype	Frequency	Case, Control Ratio Counts	Case, Control Frequencies	Chi-Square	*p*-value
Block 1	AAGTCTGATT	0.547	97.0:33.0, 89.0:121.0	0.746, 0.424	33.618	6.71 × 10^−9^ **
	CTTCGGATGC	0.421	30.0:100.0, 113.0:97.0	0.231, 0.538	31.12	2.43 × 10^−8^ *
	AAGTCTGTTC	0.024	3.0:127.0, 5.0:205.0	0.023, 0.024	0.002	0.9655
Block 2	CG	0.653	102.0:28.0, 120.0:90.0	0.785, 0.571	16.104	6.00 × 10^−5^ **
	AT	0.347	28.0:102.0, 90.0:120.0	0.215, 0.429	16.104	6.00 × 10^−5^ *
Block 3	TA	0.541	88.0:42.0, 96.0:114.0	0.677, 0.457	15.62	7.74 × 10^−5^ **
	CG	0.459	42.0:88.0, 114.0:96.0	0.323, 0.543	15.62	7.74 × 10^−5^ *

** Significant risk haplotypes associated with autistic patients. * Significant protective haplotypes associated with control subjects.

**Table 3:** List of the top 20 upregulated genes with fold changes ≥ 1.98, *p*-values ≤ 0.0025 and *q*-values ≤ 0.05.

Gene	Locus	FPKM Control	FPKM Case	log2 (Fold Change)	*p*-value	*q*-value	Ensemble Gene ID	Gene Description
*RPS12*	6:132814568–132817564	325.7	2218.21	2.76778	0.00005	0.004411	ENSG00000112306	ribosomal protein S12
*HNRNPH2*	X:101390823–101414133	79.1408	1227.64	3.95532	0.00005	0.004411	ENSG00000126945	heterogeneous nuclear ribonucleoprotein H2
*KRT8*	12:52897186–52952906	0.834316	9.49503	3.50851	0.00005	0.004411	ENSG00000170421	keratin 8
*RPL36A*	X:101390823–101414133	79.1408	1227.64	3.95532	0.00005	0.004411	ENSG00000241343	ribosomal protein L36a
*MTRNR2L8*	11:10507893–10509186	4.3216	253.717	5.87551	0.00005	0.004411	ENSG00000255823	MT-RNR2 like 8
*RPL36A-HNRNPH2*	X:101390823–101414133	79.1408	1227.64	3.95532	0.00005	0.004411	ENSG00000257529	RPL36A-HNRNPH2 readthrough
*RPL26*	17:8356901–8383213	88.4494	682.892	2.94873	0.00025	0.01343	ENSG00000161970	ribosomal protein L26
*KRBA2*	17:8356901–8383213	88.4494	682.892	2.94873	0.00025	0.01343	ENSG00000184619	KRAB-A domain containing 2
*AC135178.3*	17:8356901–8383213	88.4494	682.892	2.94873	0.00025	0.01343	ENSG00000263809	novel protein
*DBI*	2:119366920–119372560	19.2423	82.5386	2.10079	0.0004	0.017644	ENSG00000155368	diazepam binding inhibitor, acyl-CoA binding protein
*PROM1*	4:15960244–16084378	29.4262	128.084	2.12192	0.0005	0.019545	ENSG00000007062	prominin 1
*FGFBP2*	4:15960244–16084378	29.4262	128.084	2.12192	0.0005	0.019545	ENSG00000137441	fibroblast growth factor binding protein 2
*PPDPF*	20:63520764–63522206	43.8331	377.291	3.10559	0.0008	0.025989	ENSG00000125534	pancreatic progenitor cell differentiation and proliferation factor
*IGHG3*	14:105764502–105771405	26.0445	179.176	2.78233	0.00085	0.027065	ENSG00000211897	immunoglobulin heavy constant gamma 3 (G3m marker)
*CFD*	19:859663–863641	57.7465	285.781	2.30711	0.00115	0.032814	ENSG00000197766	complement factor D
*IMMP1L*	11:31369839–31509645	3.40717	39.2517	3.52611	0.0015	0.038464	ENSG00000148950	inner mitochondrial membrane peptidase subunit 1
*BAG1*	9:33218364–33264720	91.759	363.23	1.98496	0.00155	0.039018	ENSG00000107262	BAG cochaperone 1
*COX5B*	2:97646061–97648383	31.6214	128.267	2.02018	0.00165	0.040067	ENSG00000135940	cytochrome c oxidase subunit 5B
*PDZK1IP1*	1:47183581–47191044	41.5874	174.343	2.06771	0.0023	0.046077	ENSG00000162366	PDZK1 interacting protein 1
*HBQ1*	16:180458–181179	25.0227	198.741	2.98958	0.00245	0.047885	ENSG00000086506	hemoglobin subunit theta 1
*UQCRB*	8:96222946–96239149	13.8377	99.6279	2.84795	0.0025	0.048566	ENSG00000156467	ubiquinol-cytochrome c reductase binding protein

**Table 4:** List of the top 20 downregulated genes with foldchange ≤−3.5, a *p*-value ≤ 0.00005 and a *q*-value ≤ 0.0044.

Gene	Locus	FPKM Control	FPKM Case	log2 (Fold Change)	*p*-value	*q*-value	Ensemble Gene ID	Gene Description
*MPO*	17:58269854–58280935	74.2902	1.24471	−5.89929	0.00005	0.0044	ENSG00000005381	myeloperoxidase
*TMCC3*	12:94567121–94650557	377.371	6.56036	−5.84606	0.00005	0.0044	ENSG00000057704	transmembrane and coiled-coil domain family 3
*CEACAM6*	19:41708584–41786893	10.139	0.665306	−3.92976	0.00005	0.0044	ENSG00000086548	CEA cell adhesion molecule 6
*PLEKHG2*	19:39412668–39428415	65.41	4.86538	−3.74889	0.00005	0.0044	ENSG00000090924	pleckstrin homology and RhoGEF domain containing G2
*CEACAM5*	19:41708584–41786893	10.139	0.665306	−3.92976	0.00005	0.0044	ENSG00000105388	CEA cell adhesion molecule 5
*GRB10*	7:50590062–50793462	10.5363	0.725095	−3.86106	0.00005	0.0044	ENSG00000106070	growth factor receptor bound protein 10
*DDX5*	17:64498253–64662307	4447.04	280.637	−3.98607	0.00005	0.0044	ENSG00000108654	DEAD-box helicase 5
*CARS1*	11:3000921–3064707	210.99	15.4503	−3.77147	0.00005	0.0044	ENSG00000110619	cysteinyl-tRNA synthetase 1
*MMP8*	11:102711795–102727050	25.7828	0.665084	−5.27673	0.00005	0.0044	ENSG00000118113	matrix metallopeptidase 8
*AKAP1*	17:57085091–57121346	53.8928	4.44175	−3.60089	0.00005	0.0044	ENSG00000121057	A-kinase anchoring protein 1
*TMCC2*	1:205227945–205285632	96.9766	5.46508	−4.14932	0.00005	0.0044	ENSG00000133069	transmembrane and coiled-coil domain family 2
*CA1*	8:85327607–85481493	521.489	11.6803	−5.48049	0.00005	0.0044	ENSG00000133742	carbonic anhydrase 1
*ACE*	17:63477060–63521848	7.07334	0.510574	−3.7922	0.00005	0.0044	ENSG00000159640	angiotensin I converting enzyme
*ELF3*	1:201982371–202017183	9.77011	0.261935	−5.22109	0.00005	0.0044	ENSG00000163435	E74 like ETS transcription factor 3
*YPEL4*	11:57638023–57661865	15.1177	0.851345	−4.15035	0.00005	0.0044	ENSG00000166793	yippee like 4
*OLR1*	12:10158300–10191801	6.43025	0.410073	−3.97092	0.00005	0.0044	ENSG00000173391	oxidized low-density lipoprotein receptor 1
*POLE*	12:132623752–132687376	46.5435	4.11377	−3.50005	0.00005	0.0044	ENSG00000177084	DNA polymerase epsilon, catalytic subunit
*DDX41*	5:177511576–177516961	597.042	27.017	−4.46589	0.00005	0.0044	ENSG00000183258	DEAD-box helicase 41
*AC113554.1*	17:63477060–63521848	7.07334	0.510574	−3.7922	0.00005	0.0044	ENSG00000264813	novel protein
*AC243967.1*	19:41708584–41786893	10.139	0.665306	−3.92976	0.00005	0.0044	ENSG00000267881	novel protein, readthrough between CEACAM5-CEACAM6

**Table 5:** Associated KEGG pathway enrichments of the significantly up- and downregulated genes.

KEGG Pathway ID	Term Description	OGC	BGC	Strength	False Discovery Rate	Matching Proteins in Your Network (Labels)
						Upregulated genes
hsa03010	Ribosome	17	130	1.37	3.53E-16	RPS12, RPL35, RSL24D1, RPL13, RPL7, RPL21, RPS27, RPL11, RPL24, RPL34, RPL36A, RPS24, RPL29, RPL35A, RPL23, RPL26, RPS15
hsa00190	Oxidative phosphorylation	6	131	0.91	0.0041	COX5B, ATP5G3, ATP6V1G1, ATP5G2, NDUFA11, UQCRB
hsa03060	Protein export	3	23	1.37	0.0093	SRP14, IMMP1L, SEC61G
hsa05012	Parkinson’s disease	5	142	0.8	0.0242	COX5B, ATP5G3, ATP5G2, NDUFA11, UQCRB
hsa05010	Alzheimer’s disease	5	168	0.73	0.0394	COX5B, ATP5G3, ATP5G2, NDUFA11, UQCRB
						Downregulated genes
hsa04066	HIF-1 signaling pathway	9	98	0.76	0.0115	CREBBP, IGF1R, ARNT, TFRC, MKNK1, AKT2, SLC2A1, LDHA, VEGFA
hsa00620	Pyruvate metabolism	5	39	0.91	0.0243	ACSS2, ACACB, ALDH3A2, LDHA, ACACA
hsa00640	Propanoate metabolism	5	32	0.99	0.0243	ACSS2, ACACB, HIBCH, LDHA, ACACA
hsa01100	Metabolic pathways	37	1250	0.27	0.0243	EXTL3, CYP27B1,GCLC, ACSS2, ACLY, AMPD2, POMT2,ATP6V0A1, CAD, POLG, AMT, ACAD8, GLB1, SGSH, POLE, GANC, DGKA, ALAS2, PLA2G6, ACACB, PFKM, ALDH3A2, PIGN, DNMT1, HIBCH, PHGDH, ACSL6, BPGM, AMPD3, MTMR3, EARS2, PGM3, LDHA, MAN2A2, ALOX15, POLR2A, ACACA
hsa04015	Rap1 signaling pathway	11	203	0.53	0.0243	SIPA1L3, FARP2, RAPGEF2, IGF1R, SRC, AKT2, ADCY7, RASGRP3, FGFR1, DOCK4, VEGFA
hsa04152	AMPK signaling pathway	9	120	0.67	0.0243	TSC2, PFKFB4, IGF1R, TSC1, ACACB, PFKM, CRTC2, AKT2, ACACA
hsa04922	Glucagon signaling pathway	8	100	0.7	0.0243	CREBBP, ACACB, CRTC2, ITPR2, AKT2, SLC2A1, LDHA, ACACA
hsa05205	Proteoglycans in cancer	11	195	0.55	0.0243	DDX5, GAB1, IGF1R, ANK3, PTCH1, SRC, ITPR2, AKT2, FGFR1, PPP1R12B, VEGFA
hsa05211	Renal cell carcinoma	6	68	0.74	0.0289	CREBBP, GAB1, ARNT, AKT2, SLC2A1, VEGFA
hsa00061	Fatty acid biosynthesis	3	12	1.2	0.0368	ACACB, ACSL6, ACACA
hsa04910	Insulin signaling pathway	8	134	0.57	0.0401	TSC2, INPPL1, TSC1, ACACB, MKNK1, AKT2, IKBKB, ACACA
hsa01521	EGFR tyrosine kinase inhibitor resistance	6	78	0.68	0.0417	GAB1, IGF1R, SRC, AKT2, NRG1, VEGFA

BGC: background gene count; OGC: observed gene count.

**Table 6:** Clinical characteristics of patients with autism and controls from Eastern Province, Saudi Arabia.

Parameter	Control Group *n* = 132	Autism Group *n* = 70	*p*-value
Age (year)	8.04 ± 3.01	7.56 ± 3.68	0.1955
Gender	F = 71; M = 61	F = 23; M = 47	-
Weight (kg)	30.34 ± 13.36	26.40 ± 12.04	0.0582
Height (cm)	121.59 ± 22.01	121.68 ± 21.58	0.4913
Body mass index	19.93 ± 4.53	16.80 ± 1.78	<0.00001 *

The data are presented as the mean values ± standard deviations. * *p* ≤ 0.05 was considered statistically significant.

## Data Availability

Data is contained within article and Supplementary Materials.

## Supplementary Materials

The following are available online at https://www.mdpi.com/article/10.3390/ph15020158/s1, Table S1 (Supplementary Material) List of significant SNPs at *p* <0.001; Table S2 (Supplementary Material) The functional annotations of the most significant genes (p<0.0001) based on the DAVID 6.7 and Reactome.
