# Supplementary material for: Integration of Transcriptome and Exome Genotyping Identifies Significant Variants with Autism Spectrum Disorder
Source: Pharmaceuticals (Basel). 2022 Jan 27;15(2):158. doi: 10.3390/ph15020158 (PMC8880056; doi:10.3390/ph15020158)
Supplement: Supplementary file 1 [file pharmaceuticals-15-00158-s001.zip › pharmaceuticals-1520525-supplementary.pdf]

Integration of Transcriptome and Exome Genotyping Identifies Significant Variants with Autism Spectrum Disorder

Noor B. Almandil [1], Abdulla AlSulaiman [2], Sumayh A. Aldakeel [3], Deem N. Alkuroud [3], Halah Egal Aljofi [4],
Safah Alzahrani [5,6], Aishah Al-mana [5,6], Asma A Alfuraih [3], Majed Alabdali [2], Fahd A. Alkhamis [2], Sayed AbdulAzeez [3] and J. Francis Borgio [3,*]

[1] Department of Clinical Pharmacy Research, Institute for Research and Medical Consultations (IRMC), Imam Abdulrahman Bin Faisal University, Dammam 31441, Saudi Arabia; nbalmandil@iau.edu.sa
[2] Department of Neurology, College of Medicine, Imam Abdulrahman Bin Faisal University, Dammam 31441 , Saudi Arabia; aalsulaiman@iau.edu.sa (A.AlSulaiman.); mmalabdali@iau.edu.sa (M.A.); fkhamis@iau.edu.sa (F.A.A.)
[3] Department of Genetic Research, Institute for Research and Medical Consultations (IRMC), Imam Abdulrahman Bin Faisal University, Dammam 31441, Saudi Arabia; sumayh1427@gmail.com (S.A.A.); dalkroud@gmail.com (D.N.A.); asmaalfuraih95@gmail.com (A.A.A.); asayed@iau.edu.sa (S.AbdulAzeez.)
[4] Environmental Health Research Area, Institute for Research and Medical Consultations (IRMC), Imam Abdulrahman Bin Faisal University, Dammam 31441, Saudi Arabia; healjofi@iau.edu.sa
[5] Department of Mental Health, College of Medicine, Imam Abdulrahman Bin Faisal University, Dammam 31441, Saudi Arabia; sialzhrani@iau.edu.sa (S.Alzahrani.), amalmana@iau.edu.sa (A.Almana.)
[6] King Fahad Hospital of the University, Imam Abdulrahman Bin Faisal University, Dammam 34212, Saudi Arabia
* Correspondence: fbalexander@iau.edu.sa; Tel.: +966-13-3330864

Citation: Almandil, N.B.; AlSulaiman, A.; Aldakeel, S.A.; Alkuroud, D.N.; Aljofi, H.E.; Alzahrani, S.; Al-mana, A.; Alfuraih, A.A.; Alabdali, M.; Alkhamis, F.A.; et al. Integration of Transcriptome and Exome Genotyping Identifies Significant Variants with Autism Spectrum Disorder. *Pharmaceuticals* **2022**, *15*, 158. https://doi.org/10.3390/ph15020158

Academic Editor: Cinzia Ciccacci

Received: 6 December 2021
Accepted: 25 January 2022
Published: 27 January 2022

Publisher's Note: MDPI stays neutral with regard to jurisdictional claims in published maps and institutional affiliations.

Copyright: © 2022 by the authors. Licensee MDPI, Basel, Switzerland. This article is an open access article distributed under the terms and conditions of the Creative Commons Attribution (CC BY) license (https://creativecommons.org/licenses/by/4.0/).

Table S1. List of significant SNPs at $p < 0.001$.

S.No.	CHR	SNP	BP	A1	F_A	F_U	A2	CHISQ	P	OR	SE	L95	U95
1	1	exm10307	6705944	T	0.08462	0.005682	C	12.36	0.0004376	16.18	1.051	2.061	127
2	1	exm2259679	11029478	T	0.3154	0.5057	G	11.08	0.0008721	0.4503	0.2416	0.2805	0.723
3	1	exm2264801	11938050	A	0.3154	0.5244	G	12.91	0.0003268	0.4178	0.2451	0.2584	0.6755
4	1	exm2250514	20717763	A	0.5923	0.3523	C	17.38	3.06E-05	2.671	0.2382	1.675	4.261
5	1	exm27957	21795388	A	0.3308	0.5227	G	11.18	0.0008289	0.4513	0.2398	0.282	0.7221
6	1	exm2268870	23198619	G	0.1231	0.3182	T	15.82	6.97E-05	0.3008	0.3122	0.1631	0.5546
7	1	exm39585	32149721	C	0.1308	0.02841	G	11.74	0.0006114	5.145	0.523	1.846	14.34
8	1	exm2265001	37254301	G	0.4615	0.267	A	12.44	0.0004207	2.353	0.2449	1.456	3.802
9	1	exm2264831	45460832	G	0.3	0.4943	A	11.66	0.0006398	0.4384	0.2436	0.272	0.7068
10	1	exm2264832	47309265	G	0.2462	0.4318	A	11.29	0.0007809	0.4296	0.2542	0.2611	0.7071
11	1	exm59762	53320274	G	0.2308	0.4602	T	17.03	3.68E-05	0.3519	0.2573	0.2125	0.5826
12	1	exm61147	54605320	G	0.2769	0.4773	C	12.59	0.0003876	0.4195	0.2474	0.2583	0.6811
13	**1**	**exm65641**	**63999868**	**C**	**0.3952**	**0.2151**	**T**	**11.33**	**0.000762**	**2.384**	**0.2611**	**1.429**	**3.977**
14	1	exm-rs6700896	66089782	T	0.2538	0.4659	C	14.33	0.0001531	0.39	0.2519	0.238	0.6389
15	1	exm-rs1892534	66105944	A	0.2385	0.4659	G	16.61	4.60E-05	0.359	0.2553	0.2176	0.5921
16	1	exm-rs4420065	66161461	T	0.2308	0.4602	C	17.03	3.68E-05	0.3519	0.2573	0.2125	0.5826
17	1	exm-rs12753193	66169679	G	0.2385	0.4483	A	14.24	0.0001607	0.3854	0.2561	0.2333	0.6367
18	1	exm69632	77685042	C	0.3308	0.1534	T	13.33	0.000261	2.728	0.2802	1.575	4.723
19	1	exm2268717	85213900	T	0.2077	0.4375	C	17.6	2.73E-05	0.337	0.2643	0.2008	0.5657
20	1	exm2268726	95276691	C	0.3231	0.5284	T	12.79	0.0003491	0.426	0.2408	0.2657	0.6828
21	1	exm2264886	108088171	A	0.2538	0.4489	G	12.25	0.0004641	0.4177	0.2522	0.2548	0.6847
22	1	exm-rs1933182	109999838	T	0.2308	0.4302	G	13.05	0.0003038	0.3973	0.2589	0.2392	0.66
23	1	exm2268738	111369934	T	0.3254	0.5345	C	12.93	0.000324	0.4201	0.2434	0.2607	0.677
24	1	exm83649	111957097	G	0.2923	0.4943	C	12.63	0.00038	0.4225	0.2448	0.2615	0.6827
25	1	exm83663	111957370	C	0	0.1364	G	19.24	1.16E-05	0	inf	0	nan
26	1	exm83704	111957693	G	0.2923	0.5057	A	14.02	0.0001807	0.4038	0.2448	0.2499	0.6523
27	1	exm2264891	112385547	A	0.2692	0.4773	G	13.62	0.0002241	0.4035	0.2487	0.2478	0.657

28	1	exm87717	117576522	T	0.01538	0.1307	A	13.25	0.0002728	0.1039	0.7469	0.02405	0.4493
29	1	exm2259802	150163390	C	0.07692	0.2159	T	10.92	0.0009509	0.3026	0.3767	0.1446	0.6332
30	1	exm95472	150940625	G	0.2462	0.09091	T	13.63	0.0002231	3.265	0.332	1.704	6.259
31	1	exm-rs267734	150951477	G	0.2462	0.08523	A	14.89	0.0001137	3.505	0.3381	1.807	6.799
32	1	exm95523	150958836	G	0.2769	0.08046	A	20.9	4.84E-06	4.377	0.3407	2.245	8.535
33	1	exm95850	151089888	C	0.05385	0.1818	A	11.01	0.0009057	0.2561	0.435	0.1092	0.6007
34	1	exm97215	151512895	G	0.1231	0.02273	A	12.33	0.0004468	6.035	0.5719	1.967	18.51
35	1	exm103508	153954646	T	0.07692	0	C	14	0.0001832	NA	NA	NA	NA
36	1	exm109473	156551248	A	0.06923	0	G	12.55	0.0003954	NA	NA	NA	NA
37	1	exm111298	157062739	G	0.1077	0.2784	C	13.33	0.0002614	0.3128	0.3291	0.1641	0.5963
38	1	exm112941	158576394	A	0	0.09091	C	12.47	0.0004135	0	inf	0	nan
39	1	exm113021	158587368	C	0	0.125	T	17.51	2.86E-05	0	inf	0	nan
40	1	exm113223	158623092	A	0	0.09091	T	12.47	0.0004135	0	inf	0	nan
41	1	exm113224	158623103	A	0	0.09091	G	12.47	0.0004135	0	inf	0	nan
42	1	exm113458	158669980	A	0.007692	0.1307	G	15.65	7.63E-05	0.05157	1.028	0.00687	0.3871
43	1	exm113678	158746678	C	0.06923	0.2045	T	10.91	0.000954	0.2893	0.3928	0.1339	0.6247
44	1	exm2259837	164733504	G	0.03077	0.1761	A	15.6	7.83E-05	0.1485	0.5451	0.05102	0.4322
45	1	exm121603	169391154	A	0.2846	0.4716	G	10.97	0.0009279	0.4458	0.2461	0.2752	0.7222
46	1	exm-rs17346452	172053287	C	0.2231	0.07955	T	12.75	0.0003552	3.322	0.3493	1.676	6.588
47	1	exm2265060	178518008	A	0.1231	0.2898	G	12.15	0.000491	0.344	0.3145	0.1857	0.6371
48	1	exm129362	182640864	C	0.07692	0.2216	T	11.64	0.000647	0.2927	0.3759	0.1401	0.6115
49	1	exm131467	186026474	T	0.5	0.3068	C	11.74	0.0006112	2.259	0.2398	1.412	3.615
50	1	exm2264936	190551418	G	0.3692	0.5625	A	11.19	0.0008229	0.4553	0.2369	0.2862	0.7243
51	1	exm2268805	195738953	G	0.6462	0.3678	T	23.07	1.56E-06	3.139	0.2416	1.955	5.039
52	1	exm134032	197070442	T	0.3385	0.5284	G	10.91	0.0009563	0.4566	0.2391	0.2858	0.7295
53	1	exm134043	197070697	C	0.3385	0.5284	T	10.91	0.0009563	0.4566	0.2391	0.2858	0.7295
54	1	exm-rs2786098	197325908	A	0.1077	0.2727	C	12.61	0.0003847	0.3218	0.3297	0.1687	0.6141
55	1	exm2265070	197359999	G	0.2923	0.5115	A	14.7	0.0001263	0.3945	0.2453	0.2439	0.638
56	1	exm2268809	198634625	C	0.2615	0.4655	T	13.16	0.0002854	0.4066	0.2509	0.2487	0.6649
57	1	exm2250517	207535246	A	0.3231	0.5795	C	19.74	8.87E-06	0.3463	0.2418	0.2155	0.5562
58	1	exm151013	222802376	G	0.3231	0.1591	A	11.4	0.0007357	2.523	0.2786	1.461	4.356

59	1	exm151047	222803204	G	0.3231	0.1591	A	11.4	0.0007357	2.523	0.2786	1.461	4.356
60	1	exm2260036	231326086	A	0.3538	0.5682	G	13.77	0.0002068	0.4162	0.2383	0.2609	0.664
61	1	exm159168	232568041	A	0.1154	0.01705	G	13.06	0.0003016	7.522	0.6438	2.13	26.57
62	1	exm2260055	239144913	G	0.2538	0.09659	A	13.53	0.0002352	3.182	0.3252	1.682	6.018
63	1	exm163482	240492734	A	0.3154	0.1534	G	11.35	0.0007545	2.542	0.2817	1.464	4.416
64	1	exm164066	242035382	T	0	0.1176	C	16.39	5.17E-05	0	inf	0	nan
65	1	exm168221	248569674	G	0	0.09659	A	13.3	0.0002661	0	inf	0	nan
66	2	exm2269013	4905977	T	0.5635	0.3563	C	12.71	0.0003638	2.332	0.2394	1.459	3.728
67	2	exm-rs6431805	6654714	C	0.08594	0.2443	T	12.73	0.0003607	0.2908	0.3609	0.1434	0.5899
68	2	exm-rs6741819	7147973	T	0.2	0.4602	C	22.27	2.37E-06	0.2932	0.2664	0.174	0.4942
69	2	exm2254476	11500314	C	0.3846	0.5795	A	11.37	0.0007484	0.4534	0.2363	0.2854	0.7205
70	2	exm174940	20173436	G	0.1	0.005682	A	15.24	9.49E-05	19.44	1.045	2.51	150.6
71	2	exm-rs2082871	20712300	G	0.1	0.3011	A	17.88	2.35E-05	0.2579	0.3354	0.1336	0.4976
72	2	exm179384	27260469	A	0.3692	0.1882	G	12.34	0.0004432	2.524	0.2674	1.495	4.264
73	2	exm2261254	32495471	A	0.4462	0.2414	C	14.13	0.0001702	2.532	0.25	1.551	4.133
74	2	exm2261261	37603801	G	0.6154	0.3466	A	21.74	3.12E-06	3.016	0.24	1.885	4.828
75	2	exm2265315	42180912	G	0.03846	0.1648	A	12.08	0.0005101	0.2028	0.4993	0.07621	0.5395
76	2	exm-rs13429458	43638838	C	0.1538	0.3409	A	13.55	0.0002322	0.3515	0.2905	0.1989	0.6212
77	2	exm190111	44021676	T	0	0.08046	C	10.96	0.0009286	0	inf	0	nan
78	2	exm190359	44099244	T	0	0.07955	C	10.84	0.0009951	0	inf	0	nan
79	2	exm195691	58316814	G	0.5846	0.3908	A	11.21	0.0008132	2.194	0.2363	1.381	3.486
80	2	exm2265168	86135154	G	0.4615	0.2727	A	11.67	0.0006345	2.286	0.2441	1.417	3.688
81	2	exm217591	102955468	A	0.5938	0.3807	C	13.5	0.000238	2.378	0.2377	1.492	3.789
82	2	exm-rs17483466	111797458	G	0.2077	0.07386	A	11.79	0.0005971	3.287	0.3603	1.622	6.66
83	2	exm221863	113539192	A	0.06154	0	T	11.12	0.0008533	NA	NA	NA	NA
84	2	exm2254482	115848781	T	0.3308	0.5284	C	11.83	0.0005832	0.4411	0.2399	0.2756	0.7059
85	2	exm2269311	120986872	T	0.1538	0.3864	C	19.73	8.91E-06	0.2888	0.2882	0.1641	0.508
86	2	exm-rs13420028	133188106	G	0.1077	0.267	T	11.9	0.0005625	0.3313	0.3303	0.1734	0.6328
87	2	exm-rs7567288	134434824	C	0.1538	0.3239	T	11.48	0.0007045	0.3796	0.2916	0.2143	0.6723
88	2	exm2269143	142707328	T	0.3492	0.5714	G	14.25	0.0001597	0.4024	0.2434	0.2498	0.6484
89	2	exm2263953	144092133	A	0.06154	0.2069	C	12.7	0.0003656	0.2514	0.4101	0.1125	0.5616

90	2	exm232550	152322095	A	0.1538	0.3295	T	12.15	0.0004901	0.3699	0.2912	0.209	0.6546
91	2	exm232595	152331418	C	0.1538	0.3295	G	12.15	0.0004901	0.3699	0.2912	0.209	0.6546
92	2	exm2265220	153891830	G	0.1328	0.3182	A	13.96	0.0001872	0.3282	0.3066	0.1799	0.5986
93	2	exm-rs10189160	157012321	G	0.5077	0.3059	A	12.57	0.0003913	2.34	0.2418	1.457	3.759
94	2	exm234189	157425931	C	0	0.07955	A	10.84	0.0009951	0	inf	0	nan
95	2	exm234888	159663599	C	0.3231	0.5455	T	14.93	0.0001113	0.3977	0.241	0.248	0.6379
96	2	exm235173	160086654	G	0.5154	0.3182	A	12.1	0.0005053	2.279	0.2387	1.427	3.639
97	2	exm239782	169824983	C	0.1077	0.01705	T	11.71	0.0006218	6.96	0.6474	1.957	24.76
98	2	exm249736	179914640	A	0.2538	0.08523	G	16.07	6.09E-05	3.652	0.3369	1.887	7.067
99	2	exm252043	189864080	A	0.2266	0.4091	G	11.13	0.0008501	0.4231	0.2609	0.2537	0.7056
100	2	exm2269194	192458747	C	0.4846	0.2727	T	14.52	0.0001385	2.507	0.2438	1.555	4.044
101	2	exm259986	207603234	G	0.1562	0.3409	T	13.03	0.0003063	0.358	0.2908	0.2025	0.633
102	2	exm2261158	208014898	T	0.1111	0.2917	C	13.94	0.0001886	0.3036	0.3304	0.1589	0.5801
103	2	exm2265268	212807645	A	0.2937	0.5123	G	13.95	0.0001881	0.3957	0.2509	0.242	0.6471
104	2	exm-rs7590720	216898658	G	0.4692	0.2784	A	11.82	0.0005846	2.291	0.2432	1.422	3.691
105	2	exm-rs16864755	224274622	A	0.1846	0.05114	G	13.85	0.0001984	4.201	0.4101	1.881	9.386
106	2	exm272457	228493211	A	0.2077	0.3977	G	12.47	0.0004132	0.3969	0.2655	0.2359	0.6679
107	2	exm278071	235950002	A	0.2077	0.07386	G	11.79	0.0005971	3.287	0.3603	1.622	6.66
108	2	exm278779	238244963	A	0.1385	0.3125	G	12.47	0.000414	0.3536	0.3015	0.1958	0.6385
109	2	exm278798	238247734	G	0.06923	0.2273	C	13.89	0.0001942	0.2529	0.3895	0.1179	0.5426
110	3	exm-rs2727943	1897973	T	0.03846	0.2184	C	19.84	8.44E-06	0.1432	0.4916	0.05462	0.3752
111	3	exm-rs6772112	11641535	C	0.04688	0.1818	T	12.34	0.0004439	0.2213	0.4616	0.08956	0.5469
112	3	exm291396	12875443	G	0.3615	0.5625	A	12.1	0.0005031	0.4404	0.2375	0.2765	0.7015
113	3	exm292458	14187449	G	0.6	0.3693	T	15.98	6.39E-05	2.562	0.2376	1.608	4.081
114	3	exm2269605	16237372	T	0	0.125	C	17.51	2.86E-05	0	inf	0	nan
115	3	exm294619	17053499	A	0.4615	0.2273	G	18.65	1.57E-05	2.914	0.2516	1.78	4.772
116	3	exm-rs17272796	17077268	C	0.4615	0.233	T	17.67	2.63E-05	2.822	0.2505	1.727	4.611
117	3	exm2261480	27614316	C	0.5	0.25	A	20.38	6.36E-06	3	0.2471	1.848	4.869
118	3	exm2269421	27615419	T	0.6	0.3807	C	14.43	0.0001458	2.44	0.237	1.534	3.883
119	3	exm299494	37574951	A	0.5154	0.3125	G	12.84	0.0003396	2.34	0.2393	1.464	3.74
120	3	exm302016	39161456	A	0.4538	0.2443	G	14.77	0.0001213	2.57	0.2486	1.579	4.184

121	3	exm302017	39161464	A	0.4538	0.2443	G	14.77	0.0001213	2.57	0.2486	1.579	4.184
122	3	exm302175	39180263	G	0.4453	0.2616	A	11.03	0.0008945	2.266	0.2484	1.392	3.687
123	3	exm2256022	42131415	A	0.4154	0.233	G	11.62	0.000651	2.34	0.2519	1.428	3.833
124	3	exm-rs4682869	42923964	C	0.4844	0.2931	T	11.52	0.0006878	2.266	0.2429	1.407	3.647
125	3	exm324348	53899276	T	0.06154	0.1989	C	11.67	0.0006335	0.2642	0.4109	0.1181	0.5911
126	3	exm2265460	64171993	A	0.3154	0.5227	G	13.08	0.0002982	0.4206	0.2417	0.2619	0.6754
127	3	exm330361	74334560	T	0.08462	0.2557	C	14.63	0.0001305	0.2691	0.3594	0.133	0.5443
128	3	exm334983	102157365	T	0.3154	0.1534	A	11.35	0.0007545	2.542	0.2817	1.464	4.416
129	3	exm334985	102157417	G	0.3154	0.1534	A	11.35	0.0007545	2.542	0.2817	1.464	4.416
130	3	exm2269497	102240202	T	0.5968	0.3916	C	11.98	0.0005388	2.3	0.2425	1.43	3.699
131	3	exm2265485	104640080	G	0.2231	0.4148	A	12.36	0.0004376	0.4051	0.2604	0.2432	0.6749
132	3	exm-rs10433406	124192674	A	0.02308	0.1307	C	11.14	0.0008469	0.1571	0.6255	0.04612	0.5354
133	3	exm2265501	125867617	G	0.3	0.4943	A	11.66	0.0006398	0.4384	0.2436	0.272	0.7068
134	3	exm2255707	134388550	A	0.5846	0.3864	G	11.8	0.0005936	2.235	0.2359	1.408	3.549
135	3	exm357173	147121751	T	0.08462	0.2356	C	11.97	0.0005413	0.2999	0.3622	0.1474	0.6099
136	3	exm363668	169518455	C	0.3385	0.5284	T	10.91	0.0009563	0.4566	0.2391	0.2858	0.7295
137	3	exm2269688	170171738	C	0.5615	0.3466	T	14.03	0.0001794	2.414	0.2373	1.516	3.845
138	3	exm2255870	183508590	G	0.08462	0.005682	A	12.36	0.0004376	16.18	1.051	2.061	127
139	3	exm367360	183508714	A	0.06154	0	G	11.12	0.0008533	NA	NA	NA	NA
140	3	exm376221	195935346	T	0	0.1023	C	14.13	0.0001709	0	inf	0	nan
141	4	exm380211	996165	A	0.1308	0.2955	G	11.61	0.0006554	0.3587	0.3082	0.1961	0.6563
142	4	exm382553	2306259	T	0.2385	0.08046	C	14.73	0.0001241	3.579	0.3465	1.815	7.057
143	4	exm389532	10079377	T	0	0.08523	C	11.65	0.0006418	0	inf	0	nan
144	4	exm-rs7442317	29903052	A	0.3692	0.1989	G	10.98	0.0009215	2.358	0.2621	1.411	3.942
145	4	exm404236	71346701	G	0.1846	0.358	C	11.04	0.0008912	0.4061	0.2754	0.2367	0.6967
146	4	exm2256737	78734563	A	0.3154	0.5284	G	13.78	0.000205	0.4111	0.2417	0.256	0.6603
147	4	exm408162	78987157	G	0.5615	0.3125	A	19.06	1.27E-05	2.818	0.2402	1.76	4.511
148	4	exm2269935	79625240	C	0.3538	0.5455	T	11.04	0.0008928	0.4563	0.2378	0.2863	0.7273
149	4	exm2265850	82318524	A	0.6077	0.4034	G	12.49	0.00041	2.291	0.2364	1.441	3.641
150	4	exm411960	88415636	C	0.6231	0.3864	G	16.77	4.22E-05	2.625	0.2382	1.646	4.187
151	4	exm411969	88416188	T	0.6231	0.3864	G	16.77	4.22E-05	2.625	0.2382	1.646	4.187

152	4	exm2265740	106922960	A	0.3077	0.5057	G	11.97	0.0005397	0.4343	0.2431	0.2697	0.6995
153	4	exm417605	107845794	T	0.1462	0.01705	C	18.68	1.55E-05	9.871	0.6331	2.854	34.14
154	4	exm419255	113352397	G	0.5077	0.2955	A	14.22	0.000163	2.459	0.241	1.533	3.944
155	4	exm419280	113352899	G	0.5077	0.2955	A	14.22	0.000163	2.459	0.241	1.533	3.944
156	4	exm2265770	147246721	A	0.3308	0.5284	G	11.83	0.0005832	0.4411	0.2399	0.2756	0.7059
157	4	exm2265806	183777997	A	0.5615	0.3295	G	16.44	5.03E-05	2.606	0.2387	1.632	4.16
158	5	exm2261915	3311493	T	0.06154	0.2102	C	13.18	0.0002831	0.2463	0.4092	0.1105	0.5493
159	5	exm-rs7729273	7228047	T	0.2154	0.04545	C	20.8	5.10E-06	5.765	0.4201	2.53	13.13
160	5	exm2266062	23020866	G	0.3462	0.5398	A	11.29	0.0007777	0.4514	0.2385	0.2829	0.7203
161	5	exm-rs2886725	37770836	A	0.04615	0.2216	G	18.35	1.84E-05	0.17	0.4557	0.06958	0.4152
162	5	exm451684	38955796	A	0.4462	0.2273	G	16.46	4.98E-05	2.739	0.252	1.672	4.488
163	5	exm452637	41033163	C	0.007692	0.1136	T	13.13	0.0002906	0.06047	1.032	0.008006	0.4567
164	5	exm452676	41048533	T	0.3538	0.1818	C	11.65	0.0006418	2.464	0.268	1.457	4.167
165	5	exm2266081	50057729	A	0.6077	0.4148	G	11.13	0.0008486	2.186	0.236	1.376	3.471
166	5	exm459265	70308262	T	0	0.09091	C	12.47	0.0004135	0	inf	0	nan
167	5	exm459568	70818150	G	0.1774	0.05357	C	11.53	0.0006842	3.81	0.4155	1.688	8.603
168	5	exm461037	73985215	G	0.1308	0.3352	A	16.74	4.28E-05	0.2983	0.3052	0.164	0.5427
169	5	exm464559	79950781	A	0.1953	0.4318	G	18.68	1.54E-05	0.3194	0.2699	0.1882	0.5421
170	5	exm2266097	86366246	G	0.06154	0.2045	A	12.42	0.0004247	0.255	0.41	0.1142	0.5696
171	5	exm466476	89979518	T	0.06154	0.2069	C	12.7	0.0003656	0.2514	0.4101	0.1125	0.5616
172	5	exm466624	90012379	A	0.1719	0.05172	G	11.56	0.0006745	3.805	0.4148	1.688	8.579
173	5	exm2270224	91840637	T	0.1462	0.3523	G	16.32	5.35E-05	0.3147	0.2942	0.1768	0.5602
174	5	exm-rs4869419	92567539	G	0.05385	0.1932	A	12.51	0.0004047	0.2377	0.4329	0.1017	0.5553
175	5	exm-rs17376456	93557702	G	0.1077	0.2614	A	11.2	0.0008173	0.3411	0.3309	0.1783	0.6524
176	5	exm-rs17418283	94154588	C	0.4154	0.2299	T	11.99	0.0005358	2.38	0.2533	1.449	3.91
177	5	exm468894	96503523	C	0.5769	0.3693	T	12.98	0.0003141	2.329	0.2365	1.465	3.701
178	5	exm468932	96513471	C	0.1905	0.4034	G	15.44	8.51E-05	0.348	0.274	0.2034	0.5954
179	5	exm2270091	96731630	T	0.3769	0.5739	C	11.6	0.0006579	0.4492	0.2366	0.2825	0.7143
180	5	exm-rs13355008	100695179	T	0.2	0.3765	C	10.92	0.0009491	0.4141	0.2704	0.2437	0.7035
181	5	exm2270231	103670162	T	0.03846	0.2045	C	17.77	2.49E-05	0.1556	0.4929	0.0592	0.4087
182	5	exm-rs1318772	112723567	G	0.04615	0.1932	A	14.22	0.0001622	0.2021	0.4595	0.08211	0.4974

183	5	exm2270111	112823998	T	0.5923	0.3636	C	15.74	7.28E-05	2.542	0.2375	1.596	4.05
184	5	exm472550	115428381	G	0.1923	0.05682	T	13.55	0.0002322	3.952	0.3944	1.825	8.562
185	5	exm479249	135287029	T	0.5308	0.3182	C	13.99	0.0001843	2.424	0.2389	1.518	3.871
186	5	exm2266014	140522425	A	0.5154	0.2955	G	15.22	9.58E-05	2.536	0.241	1.581	4.067
187	5	exm2266018	145270339	G	0.2923	0.5227	A	16.25	5.56E-05	0.3771	0.2449	0.2334	0.6094
188	5	exm493171	148206473	G	0.1615	0.3466	C	13.05	0.0003028	0.3632	0.2862	0.2073	0.6364
189	5	exm494951	149509446	T	0.1077	0.01136	C	14	0.0001827	10.5	0.7654	2.343	47.06
190	5	exm497540	150947390	T	0	0.1136	C	15.81	7.02E-05	0	inf	0	nan
191	5	exm2257065	156671437	T	0.03077	0.1667	G	14.2	0.0001643	0.1587	0.5471	0.05432	0.4638
192	5	exm2266139	167657421	A	0.2538	0.4602	G	13.62	0.0002233	0.399	0.252	0.2435	0.6538
193	5	exm-rs11249661	179277874	A	0.4308	0.25	G	11.11	0.0008605	2.27	0.2483	1.395	3.694
194	6	exm-rs675209	7102084	T	0.2154	0.4261	C	14.87	0.000115	0.3697	0.2622	0.2211	0.618
195	6	exm2266156	11477493	A	0.5538	0.3068	G	18.84	1.42E-05	2.805	0.2405	1.75	4.494
196	6	exm-rs16883019	19969638	C	0.02308	0.1307	T	11.14	0.0008469	0.1571	0.6255	0.04612	0.5354
197	6	exm2257690	23774487	A	0.6231	0.358	G	21.1	4.37E-06	2.965	0.2397	1.853	4.743
198	6	exm523653	27277051	T	0.1692	0.3466	C	11.9	0.0005617	0.384	0.2825	0.2208	0.6681
199	6	exm523713	27279852	C	0.2	0.05682	T	14.77	0.0001217	4.15	0.3926	1.923	8.958
200	6	exm524368	27925367	A	0.06154	0.2216	G	14.73	0.0001238	0.2303	0.4076	0.1036	0.5121
201	6	exm524507	28117331	T	0.007692	0.1591	C	19.98	7.84E-06	0.04097	1.025	0.005498	0.3054
202	6	exm-rs9257819	29360183	C	0.2308	0.5398	A	29.55	5.46E-08	0.2558	0.2573	0.1545	0.4235
203	6	exm-rs2022077	29361124	A	0.2344	0.5398	T	28.55	9.14E-08	0.261	0.2577	0.1575	0.4325
204	6	exm525841	29364615	T	0.2308	0.5398	G	29.55	5.46E-08	0.2558	0.2573	0.1545	0.4235
205	6	exm525845	29364643	C	0.2308	0.5398	T	29.55	5.46E-08	0.2558	0.2573	0.1545	0.4235
206	6	exm525853	29364815	G	0.2308	0.5398	C	29.55	5.46E-08	0.2558	0.2573	0.1545	0.4235
207	6	exm525854	29364835	G	0.2308	0.5465	T	30.5	3.34E-08	0.2489	0.2584	0.15	0.4131
208	6	exm525859	29364951	A	0.2266	0.5398	G	30.1	4.10E-08	0.2498	0.2597	0.1501	0.4155
209	6	exm-rs2073149	29365423	A	0.246	0.5795	T	33.14	8.57E-09	0.2367	0.2571	0.143	0.3918
210	6	exm-rs1028411	29367399	C	0.2308	0.5398	A	29.55	5.46E-08	0.2558	0.2573	0.1545	0.4235
211	6	exm-rs2394607	29369519	C	0.2538	0.5682	T	30.07	4.18E-08	0.2586	0.2525	0.1576	0.4241
212	6	exm-rs720497	29382433	G	0.3769	0.2045	C	11.07	0.0008752	2.353	0.2601	1.413	3.917
213	6	exm-rs7770592	29395747	A	0.1846	0.3693	C	12.37	0.0004369	0.3866	0.2748	0.2257	0.6625

214	6	exm-rs1233493	29458241	C	0.06154	0	T	11	0.0009126	NA	NA	NA	NA
215	6	exm-rs734960	29475142	A	0.07692	0.2159	G	10.92	0.0009509	0.3026	0.3767	0.1446	0.6332
216	6	exm-rs1592410	29483968	A	0.5538	0.3466	G	13.07	0.0003	2.34	0.2371	1.47	3.725
217	6	exm-rs724078	29489048	G	0.3385	0.5795	A	17.42	3.00E-05	0.3712	0.2402	0.2318	0.5943
218	6	exm-rs995185	29529137	A	0.1385	0.3125	T	12.47	0.000414	0.3536	0.3015	0.1958	0.6385
219	6	exm-rs926552	29548089	T	0.06154	0	C	11.12	0.0008533	NA	NA	NA	NA
220	6	exm526267	29598286	A	0	0.1047	G	14.25	0.00016	0	inf	0	nan
221	6	exm-rs2524005	29899677	T	0.1077	0.2701	C	12.24	0.0004682	0.3261	0.3305	0.1706	0.6232
222	6	exm-rs356971	29979797	C	0.07692	0.2216	A	11.64	0.000647	0.2927	0.3759	0.1401	0.6115
223	6	exm-rs165255	29989695	G	0.1452	0.3375	C	13.66	0.0002195	0.3333	0.3049	0.1834	0.6059
224	6	exm-rs166327	30002881	G	0.3538	0.5568	A	12.36	0.0004376	0.4359	0.2381	0.2733	0.695
225	6	exm-rs259938	30008003	C	0.2154	0.4023	G	11.9	0.0005613	0.4078	0.2635	0.2433	0.6835
226	6	exm-rs1150739	30031345	G	0.3538	0.5455	A	11.04	0.0008928	0.4563	0.2378	0.2863	0.7273
227	6	exm-rs1150735	30045199	T	0.2385	0.4205	C	10.99	0.0009173	0.4316	0.2563	0.2612	0.7132
228	6	exm-rs1264709	30056476	A	0.07692	0.2299	T	12.67	0.0003718	0.2792	0.3752	0.1338	0.5824
229	6	exm-rs2523734	30129676	G	0.1923	0.375	T	11.94	0.0005483	0.3968	0.2716	0.233	0.6757
230	6	exm-rs1264569	30365320	G	0.3306	0.142	A	14.39	0.0001483	2.985	0.2951	1.674	5.324
231	6	exm-rs3130654	30823075	G	0.2462	0.09659	C	12.44	0.000421	3.054	0.3264	1.611	5.791
232	**6**	**exm-rs3130780**	**30874308**	**T**	**0.2462**	**0.07471**	**G**	**17.34**	**3.12E-05**	**4.044**	**0.353**	**2.025**	**8.077**
233	6	exm-rs2253705	30900094	A	0.3462	0.125	G	21.38	3.76E-06	3.706	0.2931	2.086	6.583
234	6	exm-rs6933400	30907176	T	0.3077	0.1193	C	16.62	4.56E-05	3.28	0.3003	1.821	5.909
235	6	exm529258	30919878	C	0.3077	0.125	G	15.45	8.49E-05	3.111	0.2967	1.739	5.566
236	6	exm-rs3095150	30932532	G	0.375	0.5852	A	13.1	0.0002951	0.4252	0.2382	0.2666	0.6783
237	6	exm-rs13191258	30978717	T	0.01538	0.1322	C	13.45	0.0002451	0.1026	0.7469	0.02373	0.4435
238	6	exm-rs3871466	30983683	G	0.06154	0.2011	A	11.94	0.0005487	0.2604	0.4111	0.1164	0.5829
239	6	exm-rs3869096	30984404	C	0.06154	0.1932	A	10.94	0.0009398	0.2739	0.4119	0.1222	0.614
240	6	exm529509	30993567	T	0.07692	0.2184	G	11.2	0.0008182	0.2982	0.3768	0.1425	0.6242
241	6	exm-rs2517550	31008368	G	0.4	0.2159	A	12.21	0.0004766	2.421	0.2562	1.465	4
242	6	exm-rs3094188	31142245	G	0.3923	0.2159	T	11.28	0.0007834	2.344	0.2566	1.418	3.876
243	6	exm-rs6921663	31154620	C	0.09231	0.2384	T	10.92	0.000953	0.3249	0.3519	0.163	0.6476
244	6	exm-rs3132496	31208610	C	0.6	0.392	A	12.95	0.0003193	2.326	0.2364	1.464	3.697

245	6	exm-rs9264942	31274380	T	0.6	0.3693	C	15.98	6.39E-05	2.562	0.2376	1.608	4.081
246	6	exm-rs2523590	31327064	G	0.1538	0.4059	A	22.43	2.18E-06	0.2661	0.2889	0.1511	0.4689
247	6	exm-rs2523586	31327435	C	0.1308	0.3011	A	12.3	0.0004527	0.3491	0.3077	0.191	0.6381
248	6	exm-rs1063632	31378510	A	0.03077	0.1989	G	19	1.31E-05	0.1279	0.5418	0.04422	0.3699
249	6	exm530708	31379109	A	0.2615	0.4886	G	16.18	5.77E-05	0.3706	0.2501	0.227	0.6052
250	6	exm530736	31382882	A	0.2154	0.4659	C	20.38	6.35E-06	0.3147	0.2614	0.1885	0.5253
251	6	exm-rs10223421	31390055	T	0.2154	0.4659	G	20.38	6.35E-06	0.3147	0.2614	0.1885	0.5253
252	6	exm-rs11752262	31431757	G	0.1	0.2841	A	15.5	8.26E-05	0.28	0.3368	0.1447	0.5418
253	6	exm-rs2395029	31431780	G	0.01538	0.125	T	12.43	0.0004226	0.1094	0.7482	0.02524	0.474
254	6	exm-rs11759988	31432861	C	0.1	0.2614	T	12.51	0.0004049	0.314	0.339	0.1616	0.6102
255	6	exm-rs2516408	31463491	T	0.2154	0.4261	C	14.87	0.000115	0.3697	0.2622	0.2211	0.618
256	6	exm-rs6916394	31464050	C	0.5	0.3103	T	11.24	0.0008026	2.222	0.24	1.388	3.557
257	6	exm-rs4713468	31470875	A	0.5079	0.3046	T	12.7	0.0003654	2.357	0.2427	1.465	3.792
258	6	exm-rs2844498	31476854	A	0.5846	0.375	G	13.21	0.0002785	2.346	0.2365	1.476	3.729
259	6	exm-rs2246618	31478986	T	0.2154	0.4261	C	14.87	0.000115	0.3697	0.2622	0.2211	0.618
260	6	exm-rs9267673	31883679	T	0.05385	0.2386	C	18.98	1.32E-05	0.1816	0.4269	0.07864	0.4192
261	6	exm-rs7746553	31895973	G	0.1	0.3068	C	18.7	1.53E-05	0.251	0.3349	0.1302	0.484
262	6	exm-rs4151664	31920873	T	0.04615	0.2102	C	16.67	4.46E-05	0.1818	0.4571	0.07421	0.4453
263	6	exm-rs592229	31930441	G	0.2769	0.1136	T	13.33	0.0002607	2.987	0.3079	1.634	5.462
264	6	exm533591	31938412	T	0.04615	0.1761	G	11.88	0.0005659	0.2263	0.4625	0.09143	0.5603
265	6	exm-rs3130349	32147696	A	0.03077	0.1477	G	11.57	0.0006715	0.1832	0.5505	0.06226	0.5388
266	6	exm535099	32185796	T	0.007692	0.1023	C	11.49	0.0007015	0.06804	1.034	0.008963	0.5166
267	6	exm-rs13209234	32415975	A	0.03906	0.1648	G	11.79	0.0005957	0.2061	0.4994	0.07743	0.5484
268	6	exm-rs3135006	32667119	A	0.2154	0.08046	G	11.38	0.0007439	3.137	0.351	1.577	6.242
269	6	exm-rs2395296	32911814	A	0.2615	0.4659	G	13.27	0.00027	0.406	0.2503	0.2486	0.6631
270	6	exm-rs209473	32922908	C	0.6154	0.3636	A	19.02	1.29E-05	2.8	0.2389	1.753	4.472
271	6	exm-rs580962	32925692	G	0.3231	0.5852	A	20.61	5.63E-06	0.3383	0.242	0.2105	0.5436
272	6	exm-rs516535	32942302	C	0.3231	0.5852	T	20.61	5.63E-06	0.3383	0.242	0.2105	0.5436
273	6	exm-rs206777	32952546	C	0.3231	0.5568	T	16.46	4.97E-05	0.3799	0.2412	0.2367	0.6095
274	6	exm-rs206776	32953711	A	0.2462	0.4545	G	13.99	0.0001835	0.3918	0.2537	0.2383	0.6443
275	6	exm-rs188245	32955976	C	0.3615	0.1932	T	10.89	0.0009676	2.365	0.2642	1.409	3.969

276	6	exm-rs172276	32959770	A	0.3462	0.1761	T	11.58	0.0006675	2.476	0.2704	1.457	4.207
277	6	exm-rs206768	32961387	C	0.3538	0.1761	T	12.54	0.0003985	2.561	0.2698	1.509	4.347
278	6	exm-rs206767	32962420	G	0.3672	0.6047	T	16.55	4.73E-05	0.3794	0.2407	0.2367	0.6081
279	6	exm-rs206765	32964696	A	0.4538	0.2386	T	15.66	7.58E-05	2.651	0.2496	1.625	4.324
280	6	exm-rs6911639	32978178	C	0.3308	0.1648	T	11.45	0.0007147	2.505	0.2757	1.459	4.301
281	6	exm-rs443623	32996350	A	0.06923	0.2216	T	13.12	0.0002916	0.2613	0.3903	0.1216	0.5615
282	6	exm540479	34850826	C	0.01538	0.1364	G	14.08	0.0001756	0.09896	0.7457	0.02295	0.4268
283	6	exm545759	41310705	T	0	0.08523	C	11.65	0.0006418	0	inf	0	nan
284	6	exm-rs9395066	45095163	C	0.3615	0.5568	A	11.44	0.0007201	0.4507	0.2374	0.283	0.7177
285	6	exm556612	54805688	C	0.4692	0.2557	A	15.06	0.0001041	2.574	0.2465	1.588	4.172
286	6	exm556646	54806489	A	0.4692	0.2557	C	15.06	0.0001041	2.574	0.2465	1.588	4.172
287	6	exm2266221	79196788	A	0.2769	0.4659	G	11.27	0.0007869	0.439	0.2475	0.2703	0.7131
288	6	exm-rs4840086	100208438	G	0.6	0.3864	A	13.68	0.0002169	2.382	0.2367	1.498	3.788
289	6	exm568818	105609468	T	0	0.142	C	20.11	7.32E-06	0	inf	0	nan
290	6	exm-rs9374227	111283592	T	0.1	0.25	C	11.1	0.0008637	0.3333	0.3403	0.1711	0.6494
291	6	exm573638	116784797	A	0.007692	0.1337	G	16.08	6.09E-05	0.05022	1.029	0.006689	0.377
292	6	exm2270399	125576372	C	0.3462	0.5625	T	14.05	0.0001783	0.4118	0.2389	0.2578	0.6577
293	6	exm2257503	126248374	C	0.3308	0.5398	T	13.19	0.0002812	0.4214	0.24	0.2633	0.6746
294	6	exm581368	137482840	G	0.1	0.01136	A	12.6	0.0003855	9.667	0.7689	2.142	43.63
295	6	exm582318	139233946	T	0.06923	0	C	12.55	0.0003954	NA	NA	NA	NA
296	6	exm582791	142691549	C	0.09231	0.2386	A	11.02	0.0009031	0.3245	0.3508	0.1631	0.6453
297	6	exm583060	143092754	A	0.01538	0.1307	G	13.25	0.0002728	0.1039	0.7469	0.02405	0.4493
298	6	exm584047	145069600	A	0.07627	0	G	12.61	0.0003833	NA	NA	NA	NA
299	6	exm584803	147136244	A	0.5403	0.3333	G	12.54	0.0003988	2.351	0.2434	1.459	3.788
300	6	exm585864	150212003	T	0.05385	0.2898	C	27.09	1.94E-07	0.1395	0.4226	0.06093	0.3193
301	6	exm585866	150212034	A	0	0.07955	G	10.84	0.0009951	0	inf	0	nan
302	6	exm2270429	150525260	C	0.5769	0.358	T	14.48	0.0001417	2.446	0.2371	1.537	3.893
303	6	exm2262071	151327848	A	0.3308	0.5909	G	20.27	6.72E-06	0.3422	0.2414	0.2132	0.5492
304	6	exm-rs7747960	152791474	A	0.1032	0.259	C	11.23	0.0008064	0.3291	0.3423	0.1682	0.6437
305	7	exm-rs10488360	4411209	A	0.5769	0.3471	G	15.75	7.23E-05	2.565	0.2397	1.604	4.104
306	7	exm-rs10499504	17561583	G	0.007692	0.125	A	14.8	0.0001194	0.05426	1.029	0.007216	0.4081

307	7	exm-rs12155172	20994491	A	0.2538	0.09091	G	14.76	0.0001221	3.402	0.3307	1.779	6.505
308	7	exm608825	21901540	C	0.02344	0.1453	T	12.89	0.0003305	0.1411	0.623	0.04162	0.4785
309	7	exm611740	29519929	G	0.2385	0.08721	A	13.12	0.0002924	3.277	0.3397	1.684	6.378
310	7	exm-rs6974491	37374510	A	0.1613	0.04706	G	10.86	0.0009837	3.894	0.4368	1.654	9.167
311	7	exm618517	44874113	T	0.3065	0.5698	C	20.12	7.29E-06	0.3336	0.2483	0.2051	0.5428
312	7	exm2264260	46463342	C	0.1231	0.3409	A	19.01	1.30E-05	0.2713	0.3107	0.1476	0.4989
313	7	exm-rs1012036	52472450	T	0.1385	0.3663	C	19.58	9.66E-06	0.2781	0.2992	0.1547	0.4998
314	7	exm2266423	69006876	A	0.5385	0.3466	G	11.24	0.0007992	2.199	0.2367	1.383	3.498
315	7	exm-rs6943555	69806023	A	0.5476	0.2989	T	18.79	1.46E-05	2.84	0.2439	1.761	4.58
316	7	exm625784	73097654	T	0.5538	0.3636	C	10.96	0.000933	2.172	0.236	1.368	3.45
317	7	exm629018	76950686	A	0.01538	0.142	C	14.91	0.0001127	0.09438	0.7446	0.02193	0.4061
318	7	exm2270768	84709356	T	0.4	0.1761	C	18.96	1.34E-05	3.118	0.2668	1.848	5.261
319	7	exm-rs1767742	86625854	C	0.4769	0.2841	A	11.98	0.0005373	2.298	0.2424	1.429	3.695
320	7	exm2266441	87220886	G	0.6016	0.3977	A	12.33	0.0004459	2.286	0.2373	1.436	3.64
321	7	exm633222	91630620	T	0.5231	0.2898	G	17.13	3.50E-05	2.688	0.2417	1.674	4.318
322	7	exm633461	91712698	G	0.5154	0.2701	A	19.1	1.24E-05	2.874	0.2448	1.778	4.644
323	7	exm2264243	91726927	C	0.5231	0.2955	A	16.25	5.54E-05	2.615	0.2411	1.63	4.195
324	7	exm2266446	91974757	A	0.5538	0.3563	G	11.78	0.0005995	2.242	0.237	1.409	3.569
325	7	exm633881	92028039	C	0.5615	0.3636	A	11.84	0.0005787	2.241	0.2362	1.411	3.561
326	7	exm634427	92733975	A	0.007692	0.1149	G	13.31	0.0002641	0.05969	1.032	0.007903	0.4508
327	7	exm634435	92734051	T	0.007692	0.1023	C	11.49	0.0007015	0.06804	1.034	0.008963	0.5166
328	7	exm639333	99669802	G	0.02308	0.1364	A	11.93	0.000553	0.1496	0.6241	0.04403	0.5083
329	7	exm-rs7385804	100235970	C	0.2462	0.4716	A	16.2	5.70E-05	0.3659	0.2535	0.2226	0.6013
330	7	exm643074	100488658	C	0.2923	0.4943	G	12.63	0.00038	0.4225	0.2448	0.2615	0.6827
331	7	exm2266456	101288516	G	0.3	0.4886	A	11.01	0.0009056	0.4485	0.2437	0.2782	0.7231
332	7	exm2270664	106642123	T	0.2538	0.4483	C	12.13	0.0004951	0.4187	0.2527	0.2552	0.6871
333	7	exm649118	107002504	C	0.1	0.01136	T	12.6	0.0003855	9.667	0.7689	2.142	43.63
334	7	exm650406	107800936	T	0.09231	0.2701	C	15.04	0.0001053	0.2748	0.3478	0.139	0.5433
335	7	exm655800	128312463	G	0.1923	0.0625	C	12.14	0.0004943	3.571	0.3827	1.687	7.562
336	7	exm655815	128315882	A	0.2969	0.1337	C	12.06	0.0005153	2.735	0.296	1.531	4.886
337	7	exm655820	128317658	T	0.3	0.142	C	11.28	0.0007842	2.589	0.2885	1.47	4.557

338	7	exm655833	128323034	G	0.3	0.142	A	11.28	0.0007842	2.589	0.2885	1.47	4.557
339	7	exm657813	130008259	G	0.007692	0.1034	A	11.64	0.0006441	0.06718	1.034	0.008849	0.5101
340	7	exm-rs1106683	131453525	A	0.1846	0.05682	G	12.36	0.0004376	3.758	0.3964	1.728	8.174
341	7	exm-rs1106684	131453665	G	0.1846	0.06024	C	11.09	0.0008668	3.532	0.3969	1.623	7.689
342	7	exm-rs802568	145959243	G	0.1923	0.0625	T	12.14	0.0004943	3.571	0.3827	1.687	7.562
343	7	exm2266575	146020936	G	0.2538	0.09659	A	13.53	0.0002352	3.182	0.3252	1.682	6.018
344	7	exm2270806	157880211	C	0.05385	0.1905	T	12.03	0.000524	0.2419	0.4354	0.103	0.5678
345	8	exm2270814	4809686	C	0.5462	0.3353	T	13.39	0.000253	2.386	0.2396	1.492	3.816
346	8	exm680383	8560602	T	0.1484	0.3239	C	12.16	0.0004875	0.3639	0.2962	0.2036	0.6504
347	8	exm2273446	17503316	G	0	0.08523	T	11.65	0.0006418	0	inf	0	nan
348	8	exm695209	36767024	C	0.2462	0.4489	T	13.29	0.0002666	0.4009	0.2538	0.2438	0.6593
349	8	exm-rs9650315	57155598	T	0.1066	0.3077	G	16.17	5.79E-05	0.2683	0.3409	0.1376	0.5234
350	8	exm-rs6995588	61003897	T	0.01538	0.1379	C	14.29	0.0001569	0.09766	0.7458	0.02264	0.4212
351	8	exm2262509	62172668	A	0.2769	0.483	C	13.28	0.0002687	0.41	0.2473	0.2525	0.6658
352	8	exm-rs16933415	68497939	C	0.1615	0.04545	T	11.74	0.0006102	4.046	0.4333	1.731	9.459
353	8	exm2266637	70954335	A	0.1692	0.3466	G	11.9	0.0005617	0.384	0.2825	0.2208	0.6681
354	8	exm2270983	71164680	G	0.2308	0.4713	T	18.5	1.70E-05	0.3366	0.2577	0.2031	0.5577
355	8	exm2270985	73963866	T	0.02308	0.142	G	12.73	0.0003598	0.1427	0.6228	0.0421	0.4836
356	8	exm2270876	78530715	T	0.3231	0.5795	C	19.74	8.87E-06	0.3463	0.2418	0.2155	0.5562
357	8	exm2266647	79417222	G	0.5077	0.233	A	24.82	6.30E-07	3.396	0.2501	2.08	5.544
358	8	exm-rs6473383	83669120	A	0.09016	0.2531	G	12.35	0.000441	0.2925	0.3641	0.1433	0.597
359	8	exm713190	101648164	G	0.1846	0.3807	C	13.76	0.0002081	0.3683	0.2742	0.2152	0.6305
360	8	exm-rs16872085	105957540	G	0.2077	0.07386	A	11.79	0.0005971	3.287	0.3603	1.622	6.66
361	8	exm715065	107773675	C	0	0.08523	T	11.65	0.0006418	0	inf	0	nan
362	8	exm718494	123966085	A	0.03077	0.1591	G	13.15	0.0002877	0.1678	0.5481	0.05731	0.4913
363	8	exm719207	124658203	C	0.03077	0.1534	T	12.35	0.0004405	0.1752	0.5493	0.0597	0.5141
364	8	exm719236	124663987	G	0.02308	0.1534	C	14.36	0.0001507	0.1304	0.6204	0.03864	0.4398
365	8	exm2258655	129384895	C	0.6077	0.3693	A	17.05	3.63E-05	2.645	0.238	1.659	4.218
366	8	exm-rs1876201	139982997	A	0.2462	0.4602	G	14.71	0.0001253	0.383	0.2536	0.233	0.6296
367	8	exm724389	142367087	T	0.2769	0.1092	C	14.13	0.000171	3.124	0.3122	1.694	5.762
368	8	exm2266705	143310815	A	0.2769	0.4886	G	13.98	0.0001848	0.4008	0.2473	0.2468	0.6508

369	8	exm727185	144672047	T	0	0.09091	C	12.47	0.0004135	0	inf	0	nan
370	8	exm731612	145603114	C	0.2692	0.4545	A	10.95	0.0009379	0.4421	0.249	0.2714	0.7203
371	8	exm732031	145639726	T	0.3538	0.5747	C	14.54	0.0001371	0.4052	0.2391	0.2536	0.6475
372	9	exm734722	334337	G	0.4453	0.2045	A	20.23	6.86E-06	3.122	0.258	1.883	5.176
373	9	exm741237	18639300	A	0	0.07955	G	10.84	0.0009951	0	inf	0	nan
374	9	exm-rs1063192	22003367	C	0.1154	0.3068	T	15.69	7.46E-05	0.2947	0.3195	0.1575	0.5512
375	9	exm-rs564398	22029547	G	0.1231	0.3011	A	13.57	0.0002293	0.3257	0.3135	0.1762	0.6021
376	9	exm-rs7865618	22031005	G	0.1231	0.3011	A	13.57	0.0002293	0.3257	0.3135	0.1762	0.6021
377	9	exm-rs2157719	22033366	G	0.1231	0.3011	A	13.57	0.0002293	0.3257	0.3135	0.1762	0.6021
378	9	exm-rs1412829	22043926	C	0.1231	0.3011	T	13.57	0.0002293	0.3257	0.3135	0.1762	0.6021
379	9	exm-rs1889899	26789940	G	0.5462	0.3239	A	15.18	9.75E-05	2.512	0.2387	1.574	4.011
380	9	exm744049	27009100	C	0.1016	0.2529	T	11.03	0.0008978	0.334	0.3406	0.1713	0.6512
381	9	exm2266809	33426434	A	0.3385	0.5398	G	12.22	0.000472	0.4362	0.2392	0.273	0.6972
382	9	exm747610	34724241	A	0.3154	0.5057	C	11.04	0.0008925	0.4502	0.2421	0.2801	0.7236
383	9	exm748413	35295880	A	0	0.08621	C	11.79	0.000596	0	inf	0	nan
384	9	exm750210	35853517	T	0.07812	0.005747	C	11.01	0.0009074	14.66	1.056	1.852	116.1
385	9	exm-rs4745062	73784264	T	0.3231	0.523	C	12.08	0.0005087	0.4353	0.2413	0.2713	0.6985
386	9	exm-rs10869758	78965376	T	0.08462	0.2273	C	10.96	0.000933	0.3143	0.3629	0.1543	0.64
387	9	exm2271094	80515456	G	0.2538	0.4489	T	12.25	0.0004641	0.4177	0.2522	0.2548	0.6847
388	9	exm760409	92003679	T	0.2308	0.08621	C	12.33	0.0004457	3.18	0.341	1.63	6.204
389	9	exm2271111	94508700	T	0.5692	0.3636	C	12.76	0.0003532	2.312	0.2365	1.455	3.676
390	9	exm765802	100684757	G	0.4538	0.267	A	11.52	0.0006871	2.281	0.2451	1.411	3.687
391	9	exm-rs755109	100696203	C	0.4462	0.2614	T	11.38	0.0007423	2.277	0.2461	1.405	3.688
392	9	exm2262583	118869883	C	0.2308	0.4148	T	11.34	0.0007598	0.4233	0.2583	0.2551	0.7023
393	9	exm779307	125391127	T	0.1385	0.3466	C	16.91	3.92E-05	0.303	0.2993	0.1685	0.5447
394	9	exm779314	125391241	A	0.1154	0.3239	G	18.06	2.14E-05	0.2723	0.3183	0.1459	0.5082
395	9	exm779568	125512908	G	0	0.07955	T	10.84	0.0009951	0	inf	0	nan
396	9	exm-rs10818918	126860510	A	0.1	0.2614	G	12.51	0.0004049	0.314	0.339	0.1616	0.6102
397	9	exm2259238	139418260	A	0.5923	0.3977	G	11.34	0.000758	2.2	0.2357	1.386	3.492
398	9	exm799291	139634495	A	0.3077	0.142	G	12.26	0.0004619	2.684	0.2876	1.528	4.717
399	10	exm-rs3136618	6001696	A	0.5538	0.3409	G	13.82	0.000201	2.4	0.2375	1.507	3.823

400	10	exm807880	6002368	G	0.5538	0.3523	T	12.34	0.0004427	2.283	0.2367	1.435	3.63
401	10	exm809549	12046546	C	0.03846	0.1882	T	15.28	9.25E-05	0.1725	0.4965	0.06519	0.4565
402	10	exm811848	16877080	C	0	0.1023	G	14.13	0.0001709	0	inf	0	nan
403	10	exm2259561	16903080	C	0.01538	0.1534	T	16.6	4.61E-05	0.08623	0.7427	0.02011	0.3697
404	10	exm2249163	24489659	G	0.4385	0.2386	A	13.64	0.0002212	2.491	0.25	1.526	4.067
405	10	exm2259572	25620487	A	0.3308	0.5227	C	11.18	0.0008289	0.4513	0.2398	0.282	0.7221
406	10	exm2259581	31817905	T	0.1803	0.4091	C	17.46	2.93E-05	0.3178	0.281	0.1832	0.5512
407	10	exm823593	50183042	A	0.1538	0.3352	G	12.84	0.0003385	0.3606	0.2908	0.2039	0.6376
408	10	exm2271288	51503335	C	0.4048	0.2235	T	11.3	0.0007735	2.362	0.2585	1.423	3.921
409	10	exm2267104	63814914	A	0.06154	0.2045	G	12.42	0.0004247	0.255	0.41	0.1142	0.5696
410	10	exm2271409	66551141	G	0.06923	0.2102	T	11.64	0.0006463	0.2794	0.3919	0.1296	0.6024
411	10	exm2271410	67974590	T	0.1016	0.2647	C	12.4	0.0004296	0.314	0.3404	0.1612	0.6119
412	10	exm830729	71874784	T	0	0.08523	C	11.65	0.0006418	0	inf	0	nan
413	10	exm-rs10999409	72332440	C	0.5077	0.2955	T	14.22	0.000163	2.459	0.241	1.533	3.944
414	10	exm-rs588517	83153246	A	0.07692	0.2386	G	13.86	0.0001967	0.2659	0.3736	0.1278	0.553
415	10	exm840916	91468925	G	0.1562	0.3276	C	11.4	0.0007356	0.3801	0.2921	0.2144	0.6739
416	10	exm841012	91487649	T	0.1587	0.3393	A	12.14	0.0004929	0.3674	0.2932	0.2068	0.6528
417	10	exm841018	91488979	T	0.1562	0.3314	A	11.8	0.0005925	0.3736	0.2924	0.2106	0.6627
418	10	exm-rs1592051	93542186	T	0.3308	0.5398	C	13.19	0.0002812	0.4214	0.24	0.2633	0.6746
419	10	exm844300	97096357	T	0.1385	0.03409	C	11.27	0.0007883	4.554	0.4869	1.754	11.82
420	10	exm853997	105793750	C	0.2923	0.1193	T	14.38	0.0001496	3.049	0.3021	1.686	5.511
421	10	exm-rs7076938	115789375	C	0.4692	0.2841	T	11.09	0.0008693	2.228	0.2425	1.385	3.584
422	10	exm859987	121436286	T	0.06154	0.1932	C	10.94	0.0009398	0.2739	0.4119	0.1222	0.614
423	10	exm2267137	124942806	G	0.2063	0.05814	A	15.04	0.0001052	4.212	0.3932	1.949	9.104
424	10	exm863476	126715692	T	0.02308	0.1307	C	11.14	0.0008469	0.1571	0.6255	0.04612	0.5354
425	10	exm2249111	132139508	T	0.3308	0.5398	C	13.19	0.0002812	0.4214	0.24	0.2633	0.6746
426	11	exm881387	4791111	C	0.2154	0.07955	T	11.65	0.0006416	3.176	0.3509	1.597	6.319
427	11	exm2271580	5193410	C	0.1154	0.2955	T	14.18	0.0001664	0.311	0.3204	0.166	0.5828
428	11	exm888199	7727886	T	0.2308	0.4375	C	14.05	0.0001778	0.3857	0.2577	0.2328	0.6392
429	11	exm2249501	10019879	A	0.1846	0.0625	G	11.01	0.0009075	3.396	0.3848	1.598	7.22
430	11	exm890236	10019879	A	0.1846	0.0625	G	11.01	0.0009075	3.396	0.3848	1.598	7.22

431	11	exm2267252	10791983	A	0.05385	0.25	G	20.71	5.33E-06	0.1707	0.4258	0.07411	0.3933
432	11	exm-rs10741657	14914878	A	0.3	0.4943	G	11.66	0.0006398	0.4384	0.2436	0.272	0.7068
433	11	exm-rs2060793	14915310	A	0.3077	0.5	G	11.36	0.0007487	0.4444	0.2426	0.2763	0.715
434	11	exm2267156	19057767	A	0.375	0.5747	G	11.77	0.0006018	0.444	0.2384	0.2782	0.7085
435	11	exm2271472	20301930	T	0.2923	0.5341	C	17.82	2.43E-05	0.3603	0.245	0.2229	0.5824
436	11	exm2264446	31037514	C	0.03125	0.1534	A	12.08	0.0005107	0.178	0.5494	0.06065	0.5225
437	11	exm2271488	37700681	T	0.4692	0.2356	G	18.21	1.98E-05	2.868	0.2506	1.755	4.687
438	11	exm901458	43876698	T	0.2462	0.4375	C	11.94	0.0005501	0.4198	0.2541	0.2552	0.6907
439	11	exm904624	47270255	T	0.2385	0.4318	C	12.29	0.0004547	0.412	0.256	0.2495	0.6804
440	11	exm-rs7120118	47286290	C	0.2385	0.4205	T	10.99	0.0009173	0.4316	0.2563	0.2612	0.7132
441	11	exm2271498	47290984	G	0.2308	0.4432	T	14.77	0.0001213	0.3769	0.2576	0.2275	0.6245
442	11	exm2249889	47298360	G	0.2692	0.4716	A	12.92	0.0003244	0.4128	0.2488	0.2535	0.6722
443	11	exm904958	47354787	T	0.2231	0.4375	C	15.18	9.75E-05	0.3692	0.2598	0.2219	0.6142
444	11	exm905210	47431703	G	0.06154	0.1932	A	10.94	0.0009398	0.2739	0.4119	0.1222	0.614
445	11	exm2267177	47454701	A	0.2385	0.4318	G	12.29	0.0004547	0.412	0.256	0.2495	0.6804
446	11	exm910257	56756664	G	0.5769	0.3864	A	10.91	0.0009579	2.166	0.2355	1.365	3.436
447	11	exm929809	66083043	T	0	0.08235	C	11.23	0.0008049	0	inf	0	nan
448	11	exm936011	68840399	A	0.2923	0.1092	G	16.38	5.19E-05	3.37	0.3103	1.834	6.19
449	11	exm939470	72946204	T	0.3615	0.1875	C	11.73	0.0006153	2.454	0.2657	1.458	4.131
450	11	exm2271624	75756145	C	0.05385	0.2159	T	15.66	7.60E-05	0.2067	0.4296	0.08905	0.4797
451	11	exm942770	76751166	A	0.07692	0.2386	G	13.86	0.0001967	0.2659	0.3736	0.1278	0.553
452	11	exm2271539	92316799	G	0.5	0.2955	T	13.25	0.0002731	2.385	0.241	1.487	3.824
453	11	exm950371	101359750	A	0.06923	0.2216	G	13.12	0.0002916	0.2613	0.3903	0.1216	0.5615
454	11	exm950533	101832590	A	0.08462	0.005747	C	12.21	0.0004761	15.99	1.051	2.037	125.5
455	11	exm950535	101832608	C	0.08462	0.005682	T	12.36	0.0004376	16.18	1.051	2.061	127
456	11	exm950539	101832670	A	0.08462	0.005747	G	12.21	0.0004761	15.99	1.051	2.037	125.5
457	**11**	**exm951212**	**102593248**	**T**	**0.2923**	**0.5057**	**C**	**14.02**	**0.0001807**	**0.4038**	**0.2448**	**0.2499**	**0.6523**
458	11	exm2249527	103779025	G	0.3615	0.5625	A	12.1	0.0005031	0.4404	0.2375	0.2765	0.7015
459	11	exm2267223	109199040	G	0.2846	0.483	A	12.27	0.0004605	0.4259	0.246	0.263	0.6899
460	11	exm957388	114576626	T	0.1615	0.3294	A	10.88	0.0009714	0.3922	0.2888	0.2227	0.6908
461	11	exm-rs490592	116298367	T	0.3077	0.142	G	12.26	0.0004619	2.684	0.2876	1.528	4.717

462	11	exm962243	119045951	T	0	0.07955	C	10.84	0.0009951	0	inf	0	nan
463	11	exm-rs2126709	123595927	T	0.007692	0.108	C	12.3	0.0004519	0.06406	1.033	0.008461	0.485
464	11	exm-rs6590330	128311059	A	0.02308	0.1591	G	15.19	9.71E-05	0.1249	0.6194	0.03708	0.4204
465	11	exm-rs1128334	128328959	A	0.02344	0.1647	G	15.63	7.68E-05	0.1217	0.6197	0.03613	0.4101
466	11	exm2271658	132320394	T	0.1385	0.3068	C	11.78	0.0005992	0.3631	0.302	0.2009	0.6563
467	11	exm972168	134234269	G	0.06923	0	A	12.55	0.0003954	NA	NA	NA	NA
468	12	exm973199	668548	A	0	0.07955	G	10.84	0.0009951	0	inf	0	nan
469	12	exm977504	6562293	T	0.1846	0.358	C	11.04	0.0008912	0.4061	0.2754	0.2367	0.6967
470	12	exm977528	6562823	T	0.2231	0.4148	C	12.36	0.0004376	0.4051	0.2604	0.2432	0.6749
471	12	exm979034	6948468	C	0.2923	0.5057	T	14.02	0.0001807	0.4038	0.2448	0.2499	0.6523
472	12	exm979360	7015707	T	0.1615	0.04545	G	11.74	0.0006102	4.046	0.4333	1.731	9.459
473	12	exm2260138	8976780	T	0.1769	0.3523	C	11.46	0.0007112	0.3952	0.2788	0.2288	0.6826
474	12	exm2267321	12578786	G	0.5615	0.3693	A	11.16	0.0008367	2.187	0.2359	1.377	3.472
475	12	exm987235	14993882	A	0	0.09195	G	12.62	0.000382	0	inf	0	nan
476	12	exm2267418	15750391	G	0.06154	0.1989	A	11.67	0.0006335	0.2642	0.4109	0.1181	0.5911
477	12	exm988079	18649057	T	0.2154	0.4138	C	13.27	0.0002698	0.3889	0.2631	0.2322	0.6513
478	12	exm988131	18719972	G	0.03125	0.1488	T	11.36	0.00075	0.1845	0.5523	0.0625	0.5447
479	12	exm2271825	19028431	C	0.5469	0.3529	T	11.17	0.0008327	2.213	0.2393	1.384	3.537
480	12	exm989495	21713402	T	0.4766	0.2443	C	17.76	2.51E-05	2.816	0.2492	1.728	4.589
481	12	exm993136	32137512	G	0.1	0.25	C	11.1	0.0008637	0.3333	0.3403	0.1711	0.6494
482	12	exm2250923	39508651	T	0.5846	0.358	C	15.5	8.27E-05	2.524	0.2375	1.585	4.021
483	12	exm2271838	43258744	T	0.1154	0.2955	C	14.18	0.0001664	0.311	0.3204	0.166	0.5828
484	12	exm-rs17291650	51213433	G	0.05385	0.2045	A	14.06	0.0001771	0.2213	0.4312	0.09506	0.5153
485	12	exm1003138	51681903	T	0.2	0.05114	C	16.36	5.25E-05	4.639	0.4064	2.092	10.29
486	12	exm1003199	51707626	T	0.2077	0.06897	C	12.8	0.0003457	3.539	0.3691	1.717	7.296
487	12	exm1006180	53044267	T	0.4231	0.2386	C	11.75	0.000609	2.34	0.2506	1.432	3.823
488	12	exm1010338	55523587	A	0.4769	0.2784	T	12.75	0.0003566	2.363	0.2431	1.467	3.806
489	12	exm1010343	55523685	T	0.4769	0.2816	C	12.24	0.0004666	2.326	0.2434	1.443	3.748
490	12	exm1010354	55523860	C	0.4769	0.2784	A	12.75	0.0003566	2.363	0.2431	1.467	3.806
491	12	exm1010375	55524172	A	0.4769	0.2784	G	12.75	0.0003566	2.363	0.2431	1.467	3.806
492	12	exm1010442	55641075	G	0.4538	0.2614	A	12.29	0.0004552	2.348	0.2459	1.45	3.803

493	12	exm1010452	55641295	G	0.4538	0.2614	A	12.29	0.0004552	2.348	0.2459	1.45	3.803
494	12	exm1010453	55641328	A	0.4538	0.2614	G	12.29	0.0004552	2.348	0.2459	1.45	3.803
495	12	exm-rs12367822	57204160	T	0.3492	0.175	G	11.36	0.0007519	2.53	0.2797	1.462	4.376
496	12	exm2271850	69842643	C	0.2538	0.4432	T	11.6	0.0006612	0.4274	0.2523	0.2607	0.7008
497	12	exm2264475	70728679	C	0.6	0.4091	A	10.9	0.0009592	2.167	0.2357	1.365	3.439
498	12	exm2271753	72609368	C	0.3077	0.5227	T	14.1	0.0001738	0.4058	0.2427	0.2522	0.6529
499	12	exm2271867	108894909	C	0.3231	0.6118	T	24.58	7.15E-07	0.3029	0.2448	0.1874	0.4894
500	12	exm1035335	109826634	A	0.3	0.1207	C	15.1	0.0001019	3.122	0.3013	1.73	5.636
501	12	exm2259971	118889488	G	0.1692	0.3977	T	18.57	1.64E-05	0.3085	0.2801	0.1782	0.5341
502	12	exm1042101	119772991	C	0	0.1023	G	14.13	0.0001709	0	inf	0	nan
503	12	exm1047380	123466304	G	0.007692	0.1023	A	11.49	0.0007015	0.06804	1.034	0.008963	0.5166
504	12	exm1048951	124352505	A	0	0.1307	T	18.37	1.82E-05	0	inf	0	nan
505	12	exm1048970	124358121	G	0	0.1307	A	18.37	1.82E-05	0	inf	0	nan
506	12	exm1052962	132549264	T	0	0.08235	C	11.23	0.0008049	0	inf	0	nan
507	13	exm1056554	23824818	A	0.2692	0.1193	G	11.24	0.000801	2.719	0.3052	1.495	4.946
508	13	exm1060035	28626716	T	0	0.07955	C	10.84	0.0009951	0	inf	0	nan
509	13	exm1064919	40229891	A	0.5312	0.2907	G	17.8	2.46E-05	2.765	0.2441	1.714	4.462
510	13	exm1064923	40229957	A	0.5308	0.2955	T	17.32	3.16E-05	2.697	0.2412	1.681	4.328
511	13	exm1065549	41835039	C	0.06923	0.2159	T	12.37	0.0004353	0.2701	0.3911	0.1255	0.5813
512	13	exm2271966	81260479	T	0.5308	0.3239	C	13.22	0.0002777	2.362	0.2384	1.48	3.768
513	13	exm-rs2762111	94051114	A	0.3308	0.5341	G	12.5	0.0004068	0.4312	0.24	0.2694	0.6901
514	13	exm2267623	106266101	A	0.09483	0.2534	G	10.87	0.0009798	0.3086	0.3696	0.1496	0.6369
515	14	exm2272151	20818131	C	0.1077	0.2898	T	14.82	0.0001184	0.2958	0.3281	0.1555	0.5627
516	14	exm2264539	22974256	A	0.2	0.3977	C	13.58	0.0002289	0.3786	0.2679	0.2239	0.6401
517	14	exm1091429	24114483	C	0.007692	0.1023	T	11.49	0.0007015	0.06804	1.034	0.008963	0.5166
518	14	exm2251731	24901433	T	0.1	0.2898	C	16.28	5.46E-05	0.2723	0.3363	0.1409	0.5264
519	14	exm2267701	30312091	A	0.3692	0.5795	G	13.23	0.0002749	0.4247	0.2374	0.2667	0.6763
520	14	exm2260309	33956209	A	0.1077	0.2614	C	11.2	0.0008173	0.3411	0.3309	0.1783	0.6524
521	14	exm2272064	33979974	C	0.5615	0.3636	T	11.84	0.0005787	2.241	0.2362	1.411	3.561
522	14	exm2260328	48997206	C	0.2734	0.4651	T	11.41	0.000732	0.4328	0.2504	0.2649	0.707
523	14	exm1100197	50583083	T	0.04762	0.2176	G	16.85	4.05E-05	0.1797	0.4578	0.07328	0.4408

524	14	exm-rs1265879	50877983	A	0.3846	0.2011	G	12.43	0.0004216	2.482	0.2613	1.487	4.142
525	14	exm2272106	70591661	C	0.3154	0.5795	T	20.96	4.69E-06	0.3342	0.2428	0.2077	0.5379
526	14	exm-rs221901	71602226	G	0.5308	0.3125	A	14.78	0.0001208	2.489	0.2394	1.556	3.979
527	14	exm1112651	73440864	C	0.5462	0.3295	T	14.39	0.0001488	2.448	0.2382	1.535	3.905
528	14	exm2251917	73440864	C	0.5462	0.3295	T	14.39	0.0001488	2.448	0.2382	1.535	3.905
529	14	exm1113028	73727509	T	0.5	0.2898	G	14.04	0.000179	2.451	0.2416	1.526	3.936
530	14	exm1113341	73964964	T	0	0.1534	C	21.87	2.91E-06	0	inf	0	nan
531	14	exm1113345	73965778	A	0	0.07955	G	10.84	0.0009951	0	inf	0	nan
532	14	exm1113408	73985794	C	0	0.1307	G	18.37	1.82E-05	0	inf	0	nan
533	14	exm1114149	74428445	A	0.5231	0.3239	G	12.28	0.0004577	2.29	0.2383	1.435	3.653
534	14	exm1114232	74489695	T	0	0.1	G	13.55	0.0002319	0	inf	0	nan
535	14	exm-rs8014204	75322794	G	0.4538	0.25	A	13.91	0.0001914	2.493	0.2477	1.534	4.051
536	14	exm2251934	75347585	C	0.6231	0.3977	A	15.19	9.72E-05	2.503	0.2376	1.571	3.988
537	14	exm1120001	89205265	C	0.03226	0.1647	T	12.97	0.0003169	0.169	0.5487	0.05767	0.4955
538	14	exm-rs17776811	90066451	A	0.3462	0.5455	C	11.95	0.0005455	0.4412	0.2385	0.2764	0.7041
539	14	exm1121075	91739081	A	0.04615	0.233	G	20.07	7.47E-06	0.1593	0.4545	0.06538	0.3883
540	14	exm2252002	93138041	C	0.5692	0.358	T	13.5	0.0002388	2.37	0.2368	1.49	3.77
541	14	exm1126941	100625902	C	0.5083	0.3125	T	10.99	0.0009157	2.275	0.2499	1.394	3.712
542	14	exm1127228	100847707	A	0.1846	0.3864	G	14.47	0.0001421	0.3596	0.274	0.2102	0.6152
543	14	exm-rs730570	101142890	G	0.1692	0.3409	A	11.23	0.000803	0.3938	0.2828	0.2262	0.6856
544	14	exm2272147	104704722	C	0.5769	0.3352	T	17.75	2.53E-05	2.704	0.2388	1.694	4.318
545	15	exm1144235	30018627	C	0.1328	0.3068	T	12.53	0.0003995	0.346	0.3075	0.1894	0.6322
546	15	exm1169547	65235776	G	0.5308	0.3409	A	11.05	0.0008854	2.187	0.237	1.374	3.48
547	15	exm1172611	68624396	A	0.04615	0.233	C	20.07	7.47E-06	0.1593	0.4545	0.06538	0.3883
548	15	exm1172631	68628163	T	0	0.09091	C	12.47	0.0004135	0	inf	0	nan
549	15	exm-rs2472299	75033400	A	0.2308	0.4148	G	11.34	0.0007598	0.4233	0.2583	0.2551	0.7023
550	15	exm-rs2472304	75044238	A	0.5462	0.3182	G	16.01	6.31E-05	2.579	0.2392	1.614	4.121
551	15	exm2267788	75052994	A	0.3308	0.5795	G	18.56	1.65E-05	0.3586	0.241	0.2236	0.575
552	15	exm-rs1378942	75077367	T	0.5462	0.3352	G	13.61	0.0002245	2.386	0.2378	1.497	3.803
553	15	exm-rs6495122	75125645	C	0.4692	0.2784	A	11.82	0.0005846	2.291	0.2432	1.422	3.691
554	15	exm1182735	81624768	C	0.1154	0.02273	T	11.02	0.0008999	5.609	0.5755	1.816	17.33

555	15	exm1183569	83499472	T	0	0.08523	C	11.65	0.0006418	0	inf	0	nan
556	15	exm1193919	101561275	T	0.1	0.005682	C	15.24	9.49E-05	19.44	1.045	2.51	150.6
557	16	exm1204207	2017804	G	0	0.07955	T	10.84	0.0009951	0	inf	0	nan
558	16	exm1204849	2070568	C	0.3923	0.2159	A	11.28	0.0007834	2.344	0.2566	1.418	3.876
559	16	exm1216236	8839954	A	0.5923	0.375	G	14.18	0.0001658	2.421	0.2368	1.522	3.852
560	16	exm2272419	10434389	C	0.1	0.2784	T	14.73	0.000124	0.288	0.3373	0.1487	0.5578
561	16	exm2252439	13090833	C	0.5769	0.3466	A	16.07	6.12E-05	2.571	0.2379	1.613	4.098
562	16	exm-rs153091	13253956	C	0.2462	0.09659	A	12.44	0.000421	3.054	0.3264	1.611	5.791
563	16	exm1222920	20441020	A	0.06923	0.2159	G	12.37	0.0004353	0.2701	0.3911	0.1255	0.5813
564	16	exm1223367	20748331	A	0.3615	0.1705	C	14.5	0.0001403	2.756	0.2711	1.62	4.688
565	16	exm1223535	20810266	C	0.2769	0.1023	T	15.69	7.45E-05	3.362	0.3167	1.807	6.254
566	16	exm1226195	23409440	A	0.1769	0.03409	G	17.78	2.48E-05	6.09	0.4747	2.402	15.44
567	16	exm1227682	24902299	G	0	0.07955	C	10.84	0.0009951	0	inf	0	nan
568	16	exm2252528	27113695	T	0.3154	0.517	C	12.4	0.0004302	0.4303	0.2416	0.268	0.6909
569	16	exm2252541	29043450	T	0.2923	0.483	G	11.31	0.0007729	0.4422	0.2448	0.2737	0.7145
570	16	exm1236395	31374535	G	0.5308	0.3125	C	14.78	0.0001208	2.489	0.2394	1.556	3.979
571	16	exm2225334	74481327	C	0.1077	0.005682	T	16.69	4.40E-05	21.12	1.042	2.74	162.8
572	16	exm1261911	81232564	T	0.1077	0.2784	G	13.33	0.0002614	0.3128	0.3291	0.1641	0.5963
573	17	exm1273421	648186	G	0.1538	0.3523	A	15.01	0.0001071	0.3343	0.2898	0.1894	0.59
574	17	exm1286675	7224921	G	0.06923	0.2159	C	12.37	0.0004353	0.2701	0.3911	0.1255	0.5813
575	17	exm1287293	7329134	A	0.3077	0.1193	G	16.62	4.56E-05	3.28	0.3003	1.821	5.909
576	17	exm1291491	8135444	G	0.1385	0.02841	C	13.03	0.0003069	5.496	0.5199	1.984	15.23
577	17	exm1291535	8138569	G	0.1385	0.02273	C	15.01	0.000107	6.911	0.5659	2.279	20.95
578	17	exm1291696	8166458	A	0.1385	0.03409	T	11.27	0.0007883	4.554	0.4869	1.754	11.82
579	17	exm1291833	8172506	G	0.2385	0.05114	T	23.09	1.55E-06	5.81	0.3993	2.656	12.71
580	17	exm2253321	8215927	T	0.1077	0.01724	C	11.53	0.0006845	6.879	0.6475	1.934	24.47
581	17	exm1292049	8224276	T	0.1769	0.3693	C	13.51	0.0002374	0.3671	0.2779	0.2129	0.6328
582	17	exm1312892	34077232	A	0.03846	0.1761	G	13.65	0.0002199	0.1871	0.4971	0.07061	0.4957
583	17	exm-rs16965350	36614563	T	0.1385	0.3182	C	13.17	0.0002842	0.3444	0.3011	0.1909	0.6214
584	17	exm1315705	36889559	T	0.2385	0.09659	C	11.38	0.0007428	2.929	0.3278	1.54	5.568
585	17	exm1315976	37054772	T	0.5154	0.3068	C	13.61	0.0002255	2.403	0.2398	1.502	3.844

586	17	exm1316097	37224211	T	0.1692	0.03409	C	16.43	5.06E-05	5.772	0.4767	2.267	14.69
587	17	exm1317944	38189659	T	0	0.09659	C	13.3	0.0002661	0	inf	0	nan
588	17	exm2252979	38243103	T	0	0.108	C	14.96	0.0001096	0	inf	0	nan
589	17	exm1338031	51901643	C	0.06154	0.1932	T	10.94	0.0009398	0.2739	0.4119	0.1222	0.614
590	17	exm1338036	51901703	C	0.05385	0.1875	G	11.75	0.000607	0.2466	0.4339	0.1054	0.5773
591	17	exm1339358	56270442	G	0.09231	0.01136	A	11.22	0.0008087	8.847	0.773	1.945	40.25
592	17	exm1348365	66364804	C	0.4692	0.2841	T	11.09	0.0008693	2.228	0.2425	1.385	3.584
593	17	exm1354846	73570718	G	0.1308	0.3068	C	13.01	0.0003106	0.3399	0.3072	0.1861	0.6207
594	17	exm1358102	74381555	A	0.3769	0.1724	G	16.18	5.76E-05	2.904	0.2702	1.71	4.932
595	17	exm1358104	74381567	G	0.0678	0.2529	A	16.38	5.17E-05	0.2148	0.4065	0.09684	0.4765
596	17	exm1358199	74387284	T	0.4692	0.2614	C	14.21	0.0001637	2.498	0.2456	1.544	4.043
597	17	exm1359079	74878259	A	0.1231	0.3693	G	23.29	1.39E-06	0.2397	0.3093	0.1307	0.4394
598	17	exm1360583	76433899	A	0.2769	0.108	G	14.48	0.0001417	3.165	0.3121	1.717	5.834
599	17	exm1364139	78272294	T	0.06154	0.2159	C	13.95	0.0001877	0.2381	0.4084	0.107	0.5302
600	17	exm-rs1046896	80685533	T	0.2538	0.4545	C	12.93	0.0003232	0.4082	0.252	0.2491	0.6691
601	17	exm1371159	81043175	T	0	0.108	G	14.96	0.0001096	0	inf	0	nan
602	18	exm-rs991014	42439886	A	0.3462	0.1591	G	14.4	0.0001475	2.798	0.2765	1.628	4.811
603	18	exm1391359	61654463	A	0.3462	0.5465	G	11.96	0.0005423	0.4393	0.2397	0.2746	0.7027
604	18	exm1392902	72343292	A	0	0.09412	T	12.92	0.0003243	0	inf	0	nan
605	19	exm1397560	830820	A	0.1308	0.01705	G	15.83	6.93E-05	8.676	0.6378	2.485	30.28
606	19	exm2253724	2079299	G	0.3462	0.5941	A	18.13	2.06E-05	0.3617	0.2416	0.2252	0.5808
607	19	exm-rs7248104	7224431	A	0.5615	0.3466	G	14.03	0.0001794	2.414	0.2373	1.516	3.845
608	19	exm1420079	8993018	A	0.1385	0.03409	G	11.27	0.0007883	4.554	0.4869	1.754	11.82
609	19	exm1420234	9003645	A	0.2769	0.09659	G	16.98	3.78E-05	3.582	0.3218	1.907	6.73
610	19	exm1422035	9086012	G	0.06154	0	T	11.12	0.0008533	NA	NA	NA	NA
611	19	exm2272761	9945354	C	0.2923	0.5227	T	16.25	5.56E-05	0.3771	0.2449	0.2334	0.6094
612	19	exm1423637	9968434	T	0.4923	0.2898	C	13.08	0.0002992	2.377	0.2416	1.48	3.816
613	19	exm1436482	15198441	T	0.04615	0.2102	G	16.67	4.46E-05	0.1818	0.4571	0.07421	0.4453
614	19	exm1436497	15198606	T	0.04615	0.2102	C	16.67	4.46E-05	0.1818	0.4571	0.07421	0.4453
615	19	exm1436499	15198631	A	0.2538	0.4489	C	12.25	0.0004641	0.4177	0.2522	0.2548	0.6847
616	19	exm1436508	15198751	C	0.04615	0.2102	T	16.67	4.46E-05	0.1818	0.4571	0.07421	0.4453

617	19	exm2272822	16808183	C	0.1769	0.4148	T	19.65	9.32E-06	0.3033	0.2761	0.1765	0.521
618	19	exm1440719	17036087	T	0.01538	0.1477	C	15.75	7.22E-05	0.09014	0.7436	0.02099	0.3872
619	19	exm1441188	17303774	T	0.6154	0.358	G	19.91	8.14E-06	2.87	0.2392	1.796	4.586
620	19	exm2272823	17958777	C	0.05385	0.1818	T	11.01	0.0009057	0.2561	0.435	0.1092	0.6007
621	19	exm1446569	19121084	C	0	0.08523	T	11.65	0.0006418	0	inf	0	nan
622	19	exm2260841	22522950	G	0.1231	0.358	A	21.54	3.47E-06	0.2517	0.3098	0.1372	0.462
623	19	exm2272797	48215633	C	0.2188	0.4034	T	11.51	0.0006935	0.4141	0.2633	0.2472	0.6938
624	19	exm1486034	48543906	C	0.07692	0.2216	G	11.64	0.000647	0.2927	0.3759	0.1401	0.6115
625	19	exm1487912	49206985	G	0.3462	0.5568	A	13.33	0.0002612	0.4214	0.2388	0.2639	0.6728
626	19	exm-rs504963	49208865	C	0.3231	0.5517	T	15.69	7.45E-05	0.3878	0.2417	0.2415	0.6227
627	19	exm1488777	49409026	A	0.06923	0	G	12.55	0.0003954	NA	NA	NA	NA
628	19	exm1494168	50905310	A	0	0.108	G	14.96	0.0001096	0	inf	0	nan
629	19	exm2253947	50912126	A	0	0.108	G	14.96	0.0001096	0	inf	0	nan
630	19	exm1494630	50971009	A	0	0.1307	C	18.37	1.82E-05	0	inf	0	nan
631	19	exm1497848	52223121	A	0.2615	0.4545	G	11.92	0.0005566	0.425	0.2505	0.2601	0.6944
632	19	exm1497888	52249672	A	0.5	0.3068	C	11.74	0.0006112	2.259	0.2398	1.412	3.615
633	19	exm-rs7507442	53278953	A	0.3615	0.6193	G	19.88	8.24E-06	0.3481	0.2396	0.2176	0.5567
634	19	exm1501784	54080067	C	0.06154	0.233	T	16.34	5.30E-05	0.2159	0.4062	0.09739	0.4787
635	19	exm-rs400322	55172578	A	0.2769	0.483	G	13.28	0.0002687	0.41	0.2473	0.2525	0.6658
636	19	exm1510830	56477710	T	0.4462	0.25	C	12.95	0.0003206	2.417	0.2479	1.487	3.928
637	19	exm1510859	56487603	A	0.5385	0.3352	G	12.67	0.0003725	2.314	0.2376	1.452	3.686
638	20	exm1517622	139456	A	0.1769	0.3636	G	12.81	0.0003447	0.3762	0.2782	0.2181	0.6489
639	20	exm1517629	139576	A	0.1769	0.3636	C	12.81	0.0003447	0.3762	0.2782	0.2181	0.6489
640	20	exm1517645	168728	T	0.1769	0.3864	A	15.7	7.43E-05	0.3414	0.2771	0.1983	0.5877
641	20	exm2254185	210061	A	0.2615	0.483	G	15.42	8.59E-05	0.3792	0.2502	0.2322	0.6191
642	20	exm-rs3761218	3776175	T	0.5846	0.3851	C	11.89	0.000563	2.248	0.2365	1.414	3.573
643	20	exm2272875	7482971	T	0.2846	0.4886	C	12.95	0.0003204	0.4164	0.246	0.2571	0.6743
644	20	exm-rs6056209	8836771	G	0.3923	0.5909	A	11.8	0.0005927	0.4469	0.2362	0.2813	0.71
645	20	exm-rs4141463	14747471	C	0.3125	0.5059	T	11.19	0.0008226	0.444	0.2447	0.2748	0.7173
646	20	exm2254184	20817745	G	0.3	0.5114	A	13.7	0.0002146	0.4095	0.2437	0.254	0.6602
647	20	exm1540821	37396120	G	0.6	0.392	A	12.95	0.0003193	2.326	0.2364	1.464	3.697

648	20	exm2268297	40783392	G	0.5769	0.3864	A	10.91	0.0009579	2.166	0.2355	1.365	3.436
649	20	exm-rs13038095	46425576	T	0.2154	0.0625	G	15.72	7.36E-05	4.118	0.3775	1.965	8.629
650	20	exm2260952	52019450	T	0.06923	0.2102	C	11.64	0.0006463	0.2794	0.3919	0.1296	0.6024
651	20	exm1554440	58576432	G	0	0.07955	A	10.84	0.0009951	0	inf	0	nan
652	20	exm2254385	61990939	A	0.2385	0.08523	G	13.75	0.0002093	3.361	0.3395	1.728	6.538
653	21	exm2254497	19220798	C	0.5846	0.3693	T	13.95	0.0001878	2.403	0.2368	1.511	3.823
654	21	exm-rs1475591	32440540	T	0.06923	0.2216	C	13.12	0.0002916	0.2613	0.3903	0.1216	0.5615
655	21	exm-rs2833256	32444485	A	0.04615	0.2069	G	16.15	5.86E-05	0.1855	0.458	0.07559	0.4551
656	21	exm1574967	44837555	G	0.0625	0.2093	A	12.64	0.0003783	0.2519	0.4104	0.1127	0.563
657	21	exm1576150	45564766	G	0.01538	0.1534	C	16.6	4.61E-05	0.08623	0.7427	0.02011	0.3697
658	21	exm1576957	45819296	C	0	0.1034	T	14.29	0.0001563	0	inf	0	nan
659	21	exm2272989	46334472	T	0.5692	0.3693	C	12.05	0.0005169	2.257	0.2361	1.421	3.585
660	22	exm1586012	19961730	A	0.007692	0.125	G	14.8	0.0001194	0.05426	1.029	0.007216	0.4081
661	22	exm2268401	20127554	G	0.2846	0.4886	A	12.95	0.0003204	0.4164	0.246	0.2571	0.6743
662	22	exm1596199	26695077	T	0.2308	0.4148	G	11.34	0.0007598	0.4233	0.2583	0.2551	0.7023
663	22	exm1598396	29913272	T	0.5615	0.358	C	12.55	0.000396	2.297	0.2366	1.445	3.652
664	22	exm1599866	30934816	T	0.1308	0.3125	C	13.72	0.0002117	0.331	0.3068	0.1814	0.6039
665	22	exm1606293	37906262	T	0.3308	0.1529	C	13.15	0.0002869	2.737	0.2831	1.572	4.768
666	22	exm-rs9607469	37919267	A	0.3	0.1307	G	13.27	0.00027	2.851	0.2944	1.601	5.076
667	22	exm1614469	43024271	C	0.007692	0.108	G	12.3	0.0004519	0.06406	1.033	0.008461	0.485
668	22	exm-rs9627183	48284514	A	0.007692	0.1023	G	11.49	0.0007015	0.06804	1.034	0.008963	0.5166
669	22	exm1622259	50752150	T	0.3538	0.1552	C	16.1	6.02E-05	2.981	0.2784	1.728	5.145
670	23	exm2268600	80201432	G	0.5465	0.2963	A	13.78	0.0002056	2.862	0.2871	1.63	5.025
671	23	exm2273280	95738501	C	0.4706	0.25	T	11.43	0.0007235	2.667	0.294	1.499	4.745
672	23	exm2273167	95794612	T	0.4353	0.2059	C	13.26	0.0002711	2.973	0.3047	1.636	5.402
673	23	exm1655810	122536945	A	0	0.1324	G	12.39	0.0004324	0	inf	0	nan
674	23	exm-rs5904726	146322623	G	0.1294	0.3529	A	13.36	0.0002577	0.2725	0.3696	0.1321	0.5624
675	23	exm1666749	153762634	A	0.03529	0.2206	G	14.14	0.0001698	0.1293	0.6231	0.03811	0.4384
676	24	exm-rs1865680	6868118	A	0	0.375	G	19.68	9.15E-06	0	inf	0	nan
677	24	exm-rs2032624	15026424	C	0	0.3421	A	16.42	5.07E-05	0	inf	0	nan
678	24	exm-rs2032658	15581983	G	0	0.3421	A	17.52	2.84E-05	0	inf	0	nan

679	24	exm-rs2032631	21867787	A	0.02273	0.375	G	16.86	4.02E-05	0.03876	1.063	0.004826	0.3113
680	25	exm1667537	91133892	G	0	0.08523	T	11.65	0.0006418	0	inf	0	nan
681	25	exm1667541	91134179	T	0	0.08523	G	11.65	0.0006418	0	inf	0	nan
682	26	exm2263340	8701	G	0.125	0.4713	A	20.17	7.09E-06	0.1603	0.4347	0.06837	0.3758

CHR: Chromosome; SNP: Illumina single nucleotide ID; BP: Base pair position at the respective chromosome as per GRCh37.p13; A1: Minor allele name; F_A: Frequency of minor allele in cases; F_U: Frequency of minor allele in controls; A2: Major allele name; CHISQ: Basic allelic test chi-square; P: *p*-value; OR: Odd ratio; SE: Standard error; L95: Lower bound of 95% confidence interval for odds ratio; U95: Upper bound of 95% confidence interval for odds ratio.

Table S2. The functional annotations of the most significant genes (p<0.0001) based on the DAVID 6.7 and Reactome.

ID	Gene Name	GAD_DISEASE	UP_SEQ_FEATURE
CD55	*CD55 molecule (Cromer blood group)(CD55)*	Birth Weight|Leukemia|Leukemia, Myeloid, Acute|Precursor Cell Lymphoblastic Leukemia-Lymphoma,Breath Tests,Chorioamnionitis|Fetal Membranes, Premature Rupture|Infection of amniotic sac and membranes,Chorioamnionitis|Fetal Membranes, Premature Rupture|Infection of amniotic sac and membranes|Obstetric Labor, Premature|Pre-Eclampsia|Premature Birth,Coronary Artery Disease,diabetes, type 1,Down Syndrome|,Lymphoma, Non-Hodgkin,macular degeneration,Meningeal Neoplasms|meningioma,null,	chain:Complement decay-accelerating factor,compositionally biased region:Ser/Thr-rich,disulfide bond,domain:Sushi 1,domain:Sushi 2,domain:Sushi 3,domain:Sushi 4,glycosylation site:N-linked (GlcNAc...),helix,lipid moiety-binding region:GPI-anchor amidated serine,modified residue,propeptide:Removed in mature form,sequence conflict,sequence variant,signal peptide,splice variant,strand,turn,
CTR9	*CTR9 homolog, Paf1/RNA polymerase II complex component(CTR9)*	Coronary Disease,Osteoporosis,	chain:RNA polymerase-associated protein CTR9 homolog,compositionally biased region:Lys-rich,compositionally biased region:Poly-Gly,compositionally biased region:Poly-Lys,compositionally biased region:Ser-rich,modified residue,repeat:TPR 1,repeat:TPR 10,repeat:TPR 11,repeat:TPR 12,repeat:TPR 13,repeat:TPR 14,repeat:TPR 15,repeat:TPR 16,repeat:TPR 2,repeat:TPR 3,repeat:TPR 4,repeat:TPR 5,repeat:TPR 6,repeat:TPR 7,repeat:TPR 8,repeat:TPR 9,

H2AFV	*H2A histone family member V(H2AFV)*		chain:Histone H2A.v,cross-link:Glycyl lysine isopeptide (Lys-Gly) (interchain with G-Cter in ubiquitin),modified residue,sequence variant,
HCP5	*HLA complex P5 (non-protein coding)(HCP5)*	Acquired Immunodeficiency Syndrome|HIV Infections,Acquired Immunodeficiency Syndrome|HIV Seropositivity,AIDS progression,Drug Hypersensitivity|HIV Infections|[X]Human immunodeficiency virus disease,drug-induced liver injury (flucloxacillin),HIV Infections,HIV-1 control,Hypersensitivity,Lupus Erythematosus, Systemic,	chain:HLA class I histocompatibility antigen protein P5,sequence conflict,sequence variant,

MICA	*MHC class I polypeptide-related sequence A(MICA)*	Abortion, Spontaneous,Addison Disease|Addison's disease,Addison Disease|Addison's disease|Autoimmune Diseases,Adenocarcinoma|Adenosquamous carcinoma|Carcinoma, Adenosquamous|Carcinoma, Squamous Cell|Cervical Neoplasm|Squamous cell carcinoma|Uterine Cervical Neoplasms,Adenocarcinoma|Stomach Neoplasms,Arthritis, Juvenile Rheumatoid|Chronic Childhood Arthritis,Arthritis, Rheumatoid|Rheumatoid Arthritis|Rheumatoid spondylitis|Spondylitis, Ankylosing,Autoimmune Diseases|Diabetes, Gestational|Gestational diabetes mellitus ,Autoimmune Diseases|Mixed Connective Tissue Disease,Behcet Syndrome,Behcet Syndrome|Inflammatory bowel disease, NOS|Inflammatory Bowel Diseases,Bile Duct Neoplasms|Cholangiocarcinoma|Cholangitis, Sclerosing,Breast Cancer,Brucellosis,Carcinoma, Squamous Cell|Mouth Neoplasms|Squamous cell carcinoma,Carcinoma|Nasopharyngeal Neoplasms,Celiac disease,Celiac Disease|Diabetes Mellitus, Insulin-Dependent|Diabetes Mellitus, Type 1,Cervical Neoplasm|Hypersensitivity|Uterine Cervical Neoplasms,Cervical Neoplasm|Papillomavirus Infections|Tumor Virus Infections|Uterine Cervical Neoplasms,Cholelithiasis,Chronic ulcerative colitis|Colitis, Ulcerative,Chronic ulcerative colitis|Colitis, Ulcerative|Crohn Disease|Crohn's disease|Inflammatory bowel disease, NOS|Inflammatory Bowel Diseases|Peripheral Vascular Diseases,Colorectal Cancer,Diabetes Mellitus, Insulin-Dependent|Diabetes Mellitus, Type 1,Diabetes Mellitus, Type 1,Giant Cell Arteritis|Temporal Arteritis,Graves Disease|Graves' Disease,HIV Infections|[X]Human immunodeficiency virus disease,Inflammatory bowel disease, NOS|Inflammatory Bowel Diseases,Lupus Erythematosus, Systemic|Systemic lupus erythematosus,melanoma|Uveal Neoplasms,Multiple Sclerosis,normal variation,Oral Submucous Fibrosis,Psoriasis,Rheumatoid spondylitis|Spondylitis, Ankylosing,Type 2 Diabetes| edema | rosiglitazone,	
ANXA 9	*annexin A9(ANXA9)*	melanoma,	chain:Annexin A9,modified residue,repeat:Annexin 1,repeat:Annexin 2,repeat:Annexin 3,repeat:Annexin 4,sequence variant,

BRD2	*bromodomain containing 2(BRD2)*	Abortion, Spontaneous,Alzheimer's disease ,Asthma,Azoospermia,Heart Rate,Juvenile Myoclonic Epilepsy,Lupus Erythematosus, Systemic,null,photoparoxysmal response,Triglycerides,	chain:Bromodomain-containing protein 2,compositionally biased region:Arg/Lys-rich (highly basic),compositionally biased region:Glu/Ser-rich,compositionally biased region:Poly-Glu,compositionally biased region:Poly-Lys,compositionally biased region:Poly-Pro,compositionally biased region:Poly-Ser,compositionally biased region:Ser-rich,domain:Bromo 1,domain:Bromo 2,domain:ET,helix,modified residue,mutagenesis site,sequence variant,short sequence motif:Nuclear localization signal,splice variant,turn,
CDC14C	*cell division cycle 14C, pseuodgene(CDC14C)*	Calcium,Cholesterol,Glucose,Heart Failure,Parkinson Disease,Platelet Aggregation,Sleep,Triglycerides,	
CCDC88C	*coiled-coil domain containing 88C(CCDC88C)*	Body Mass Index,Insulin,Insulin Resistance,Waist Circumference,	chain:Protein Daple,modified residue,region of interest:DVL1-binding,sequence conflict,sequence variant,short sequence motif:PDZ-binding,splice variant,
DOCK8	*dedicator of cytokinesis 8(DOCK8)*	Heart Failure,Platelet Count,Tobacco Use Disorder,	chain:Dedicator of cytokinesis protein 8,domain:DHR-1,domain:DHR-2,modified residue,sequence conflict,sequence variant,splice variant,

ITGA11	*integrin subunit alpha 11(ITGA11)*	ADHD | attention-deficit hyperactivity disorder,Attention Deficit Disorder with Hyperactivity,Cell Adhesion Molecules,Coronary Disease,Electrocardiography,lung cancer ,Psychomotor Performance,Survival,Tobacco Use Disorder,	chain:Integrin alpha-11,compositionally biased region:Poly-Arg,compositionally biased region:Poly-Leu,disulfide bond,domain:VWFA,glycosylation site:N-linked (GlcNAc...),mutagenesis site,repeat:FG-GAP 1,repeat:FG-GAP 2,repeat:FG-GAP 3,repeat:FG-GAP 4,repeat:FG-GAP 5,repeat:FG-GAP 6,repeat:FG-GAP 7,sequence variant,signal peptide,topological domain:Cytoplasmic,topological domain:Extracellular,transmembrane region,
HLA-DMA	*major histocompatibility complex, class II, DM alpha(HLA-DMA)*	Acquired Immunodeficiency Syndrome|HIV Seropositivity,antiphospholipid syndrome,Arthritis, Rheumatoid,Body Composition,condyloma acuminata,Dermatitis, Contact|,Heart Rate,HIV,lupus erythematosus,Lupus Erythematosus, Systemic,measles,medicamentosa like dermatitis, trichloroethylene-induced,Multiple Sclerosis,multiple sclerosis; IgA nephropathy,normal variation,Psoriasis,rheumatoid arthritis,systemic lupus erythematosus,Type 2 Diabetes| edema | rosiglitazone,	chain:HLA class II histocompatibility antigen, DM alpha chain,disulfide bond,domain:Ig-like C1-type,glycosylation site:N-linked (GlcNAc...),helix,region of interest:Alpha-1,region of interest:Alpha-2,region of interest:Connecting peptide,sequence variant,signal peptide,strand,topological domain:Cytoplasmic,topological domain:Lumenal,transmembrane region,turn,
MGAT5B	*mannosyl (alpha-1,6-)-glycoprotein beta-1,6-N-acetyl-glucosaminyltransferase, isozyme B(MGAT5B)*	Hemoglobin A, Glycosylated,Lipoproteins, LDL,Tobacco Use Disorder,Type 2 Diabetes| edema | rosiglitazone,	chain:Alpha-1,6-mannosylglycoprotein 6-beta-N-acetylglucosaminyltransferase B,glycosylation site:N-linked (GlcNAc...),sequence variant,splice variant,topological domain:Cytoplasmic,topological domain:Lumenal,transmembrane region,
MTHFD1L	*methylenetetrahydrofolate dehydrogenase (NADP+ dependent) 1-like(MTHFD1L)*	Acquired Immunodeficiency Syndrome|Disease Progression,Alzheimer Disease,Alzheimer's Disease,Alzheimer's disease ,Aorta,Body Mass Index,Body Weight,Cleft Lip|Cleft Palate|Tooth Abnormalities,Coronary Artery Disease,Coronary Artery Disease|Diabetes Mellitus, Type 1|Diabetic Nephropathies,Coronary Artery Disease|Myocardial Infarction,Coronary Disease,neural tube defects ,Respiratory Function Tests,Stroke,Tunica Media,Type 2 Diabetes| edema | rosiglitazone,	chain:Monofunctional C1-tetrahydrofolate synthase, mitochondrial,compositionally biased region:Poly-Gly,modified residue,nucleotide phosphate-binding region:ATP,region of interest:Formyltetrahydrofolate synthetase,region of interest:Methylenetetrahydrofolate dehydrogenase and cyclohydrolase,sequence conflict,sequence variant,splice variant,transit peptide:Mitochondrion,

MYO9B	*myosin IXB(MYO9B)*	Celiac disease,celiac disease lupus erythematosus rheumatoid arthritis,Celiac Disease|,Celiac Disease|Dermatitis Herpetiformis|Inflammatory Bowel Diseases,Celiac Disease|Down Syndrome,Colitis, Ulcerative|Crohn Disease|Inflammatory Bowel Diseases,coronary spastic angina,Crohn Disease,Crohn's disease ulcerative colitis,Diabetes Mellitus, Insulin-Dependent|Diabetes Mellitus, Type 1,diabetes, type 1 ,Multiple Sclerosis,Schizophrenia,	chain:Myosin-IXb,domain:IQ 1,domain:IQ 2,domain:IQ 3,domain:IQ 4,domain:Myosin head-like,domain:Ras-associating,domain:Rho-GAP,modified residue,nucleotide phosphate-binding region:ATP,region of interest:Actin-binding,region of interest:Neck or regulatory domain,region of interest:Tail,sequence conflict,splice variant,zinc finger region:Phorbol-ester/DAG-type,
OR12D2	*olfactory receptor family 12 subfamily D member 2 (gene/pseudogene)(OR12D2)*	female preferences for male odors,Lipoproteins, VLDL,Lupus Erythematosus, Systemic,	chain:Olfactory receptor 12D2,disulfide bond,glycosylation site:N-linked (GlcNAc...),sequence variant,topological domain:Cytoplasmic,topological domain:Extracellular,transmembrane region,
OR5V1	*olfactory receptor family 5 subfamily V member 1(OR5V1)*	female preferences for male odors,HIV Infections|[X]Human immunodeficiency virus disease,Lupus Erythematosus, Systemic,	chain:Olfactory receptor 12D3,chain:Olfactory receptor 5V1,disulfide bond,glycosylation site:N-linked (GlcNAc...),sequence conflict,sequence variant,topological domain:Cytoplasmic,topological domain:Extracellular,transmembrane region,
PFAS	*phosphoribosylformylglycinamidine synthase(PFAS)*	Tobacco Use Disorder,	
RAET1E	*retinoic acid early transcript 1E(RAET1E)*	normal variation,	chain:NKG2D ligand 4,compositionally biased region:Poly-Ser,disulfide bond,glycosylation site:N-linked (GlcNAc...),region of interest:MHC class I alpha-1 like,region of interest:MHC class I alpha-2 like,sequence conflict,sequence variant,signal peptide,splice variant,topological domain:Cytoplasmic,topological domain:Extracellular,transmembrane region,

RNF144A	*ring finger protein 144A(RNF144A)*	Amyotrophic Lateral Sclerosis,Body Fat Distribution,Cholesterol, LDL,Depressive Disorder, Major,Immunoglobulin E,Iron,longevity,Perphenazine,response to antipsychotic treatment,Schizophrenia,Stroke,Tobacco Use Disorder,	chain:Probable E3 ubiquitin-protein ligase RNF144A,helix,sequence variant,strand,transmembrane region,turn,zinc finger region:IBR-type,zinc finger region:RING-type 1; atypical,zinc finger region:RING-type 2; degenerate,
SLC8A3	*solute carrier family 8 member A3(SLC8A3)*	Cystic Fibrosis,Iron,Tobacco Use Disorder,	chain:Sodium/calcium exchanger 3,domain:Calx-beta 1,domain:Calx-beta 2,glycosylation site:N-linked (GlcNAc...),region of interest:Calmodulin-binding,repeat:Alpha-1,repeat:Alpha-2,sequence variant,signal peptide,splice variant,topological domain:Cytoplasmic,topological domain:Extracellular,transmembrane region,
SFTA2	*surfactant associated 2(SFTA2)*	Behcet Syndrome,Lupus Erythematosus, Systemic,	chain:Surfactant-associated protein 2,glycosylation site:N-linked (GlcNAc...),sequence variant,signal peptide,
ZNF600	*zinc finger protein 600(ZNF600)*	Phospholipids,	chain:Zinc finger protein 600,modified residue,sequence conflict,sequence variant,zinc finger region:C2H2-type 10,zinc finger region:C2H2-type 11; degenerate,zinc finger region:C2H2-type 12,zinc finger region:C2H2-type 13,zinc finger region:C2H2-type 14,zinc finger region:C2H2-type 15,zinc finger region:C2H2-type 16,zinc finger region:C2H2-type 17,zinc finger region:C2H2-type 18,zinc finger region:C2H2-type 19,zinc finger region:C2H2-type 1; degenerate,zinc finger region:C2H2-type 2,zinc finger region:C2H2-type 20,zinc finger region:C2H2-type 3,zinc finger region:C2H2-type 4,zinc finger region:C2H2-type 5,zinc finger region:C2H2-type 6,zinc finger region:C2H2-type 7,zinc finger region:C2H2-type 8,zinc finger region:C2H2-type 9,

ZKSCAN8	*zinc finger with KRAB and SCAN domains 8(ZKSCAN8)*		chain:Zinc finger protein 192,domain:KRAB,domain:SCAN box,modified residue,sequence conflict,sequence variant,zinc finger region:C2H2-type 1,zinc finger region:C2H2-type 2,zinc finger region:C2H2-type 3,zinc finger region:C2H2-type 4,zinc finger region:C2H2-type 5,zinc finger region:C2H2-type 6,zinc finger region:C2H2-type 7,zinc finger region:C2H2-type 8,zinc finger region:C2H2-type 9,
